# LMO3 promotes hepatocellular carcinoma invasion, metastasis and anoikis inhibition by directly interacting with LATS1 and suppressing Hippo signaling

**DOI:** 10.1186/s13046-018-0903-3

**Published:** 2018-09-15

**Authors:** Yang Cheng, Tianlu Hou, Jian Ping, Tianyang Chen, Baobing Yin

**Affiliations:** 1 Shuguang Hospital Affiliated to Shanghai University of Traditional Chinese Medicine, Shanghai, 201203 China; 2 Department of General Surgery, Huashan Hospital, Fudan University, Shanghai, 200040 China

**Keywords:** LMO3, Hepatocellular carcinoma, Invasion, Metastasis, Hippo signaling

## Abstract

**Background:**

In this research, we aimed to investigate the biological functions of LIM domain only 3 (LMO3) in hepatocellular carcinoma (HCC) and uncover the underlying molecular mechanism in it.

**Methods:**

HCC tissue microarray (*n* = 180) was used to analyze the correlation between LMO3 expression and clinicopathological findings. In vitro transwell matrigel invasion assay and annexin V anoikis assay in HCC cells were conducted to investigate LMO3 related biological functions. In vivo intrahepatic and lung metastasis models were used to determine the role of LMO3 in HCC metastasis. Quantitative real-time PCR, western blotting and immunohistochemical staining were performed to investigate the expression and mechanism of LMO3 in HCC.

**Results:**

We found that the expression of LMO3 was significantly upregulated in HCC tissues, and it was closely related to clinicopathological findings and patient prognoses. Knockdown of LMO3 suppressed the invasion and anoikis inhibition of HCC cells in vitro. Meanwhile, the metastasis of SMMC-7721 cells was also suppressed by LMO3 knockdown in vivo. Furthermore, we found that LMO3 knockdown increased the phosphorylation of YAP and LATS1, and decrease Rho GTPases activities. LMO3 directly interacted with LATS1, and thus suppressed Hippo signaling. Recombinant LMO3 (rLMO3) protein administration decreased the phosphorylation of YAP and LATS1, and increased Rho GTPases activities. The inhibitors of the Hippo pathway abrogated rLMO3 protein-induced HCC cell invasion and anoikis inhibition.

**Conclusions:**

These results suggest that LMO3 promotes HCC cell invasion and anoikis inhibition by interacting with LATS1 and suppressing Hippo signaling. LMO3 may serve as a potential therapeutic target for HCC in future.

**Electronic supplementary material:**

The online version of this article (10.1186/s13046-018-0903-3) contains supplementary material, which is available to authorized users.

## Background

Hepatocellular carcinoma (HCC) is one of the most common malignancies and a leading cause of cancer-related mortality worldwide [1, 2]. The risk factors for HCC, such as hepatitis B virus or hepatitis C virus infection, are well documented. Tumor metastasis are the main cause of death in patients with HCC. Recent studies suggest that tumor metastasis is a complex process affected by multiple procedures and multiple mediators in human cancers [3, 4]. However, the underlying molecular mechanism in HCC metastasis still remains poorly understood. Tumor metastasis may be affected by many intracellular signaling molecules and extracellular components, such as cytokines, neurotransmitters or the extracellular matrix [5, 6].

The Hippo pathway is an evolutionarily conserved signaling module that plays critical roles in liver size control and tumorigenesis [7, 8]. In mammals, the Hippo pathway is a kinase cascade, wherein Macrophage Stimulating 1/2 (MST1/2), in a complex with its regulatory protein Salvador Family WW Domain Containing Protein 1 (SAV1), phosphorylates and activates Large Tumor Suppressor Kinase 1/2 (LATS1/2). Yes-Associated Protein (YAP)/ Tafazzin (TAZ) can be phosphorylated and inactivated by active LATS1/2; when it is restrained in the cytoplasm, it loses its transcriptional activation of pro-proliferation and anti-apoptosis genes. The Hippo pathway can be activated by many biological factors including contact inhibition, mechanical strain on the cell, cell polarity/adhesion molecules, other signaling pathways and cellular metabolic status [9]. Cytokinesis failure has recently been shown to trigger the Hippo pathway [10]. Nevertheless, the link between LIM domain only 3 (LMO3) and the Hippo pathway has not been reported.

The LMO protein family includes four members: LMO1, LMO2, LMO3 and LMO4. Although LMO proteins lack DNA binding activity, some reports indicate that they are involved in the transcriptional regulation of target genes in collaboration with other transcription factors. Genetic analyses indicate that LMO1 and LMO2 contribute to the genesis of immature and aggressive T-cell leukemia [11]. LMO4 was implicated in the development of breast cancer [12, 13]. LMO3 was reported to form a complex with neuronal-specific basic helix-loop-helix (bHLH) transcription factor Helix-Loop-Helix protein 2 (HEN2), which was also expressed at higher levels in unfavorable neuroblastoma than in the favorable type. Moreover, LMO3 has been reported to play important roles in some types of cancer, including neuroblastoma [14, 15] and lung cancer [16, 17].

In this research, we found that the expression of LMO3 was significantly upregulated in HCC tissues. LMO3 expression was closely related to clinicopathological findings or patient prognoses. Knockdown of LMO3 suppresses the invasion, metastasis and anoikis inhibition of HCC cells. Further, the effects of LMO3 on the biological behaviors of HCC cells are dependent on the suppression of Hippo signaling.

## Methods

### Cell culture

Human HCC cell lines, including HCCLM3, HepG2, Huh-7, MHCC-97H, MHCC-97 L, SK-Hep1, SMMC-7721, SNU-423 and SNU-449 were purchased from Cell Bank of the Chinese Academy of Sciences. Dulbecco’s modified Eagle’s medium (DMEM) contained 10% (*v*/v) fetal calf serum (FCS) and 1% antibiotics was used. Cells were incubated at 37 °C in a humidified incubator under 5% CO_2_ condition.

### Clinical samples

Clinical human HCC (16 cases) and corresponding non-cancerous liver (CNL) tissues (12 cases), in which 12 cases were paired, were obtained from Shanghai University of Traditional Chinese Medicine Affiliated Shuguang Hospital. Additionally, Human tissue microarray contained 180 cases of HCC samples was bought from Alenabio.

### Ethics, consent and permissions

All human samples were obtained with informed consent. The protocols were approved by the ethical review committee of the World Health Organization Collaborating Center for Research in Human Production (authorized by the Shanghai Municipal Government).

### Quantitative real-time PCR

Total RNA was extracted by Trizol (Takara), and reversely transcribed by PrimeScript RT-PCR kit (Perfect Real Time). Quantitative real-time PCR analyses were performed by SYBR *Premix Ex Taq* (Takara) on a 7500 real-time PCR system (Applied Biosystems), with recommended thermal cycling settings: one initial cycle at 95 °C for 30 s followed by 40 cycles of 5 s at 95 °C and 31 s at 60 °C. Primer sequences used for human LMO3, CTGF, ANKRD1 and CYR61 detection were shown in Additional file 1: Table S1.

### Immunohistochemical staining

All tissue samples were fixed in phosphate-buffered neutral formalin, embedded in paraffin, and cut into 5 μm thick sections. The sections were deparaffinized and rehydrated, incubated with 0.3% hydrogen peroxide/phosphate-buffered saline for 30 min, and blocked with 10% BSA (Sangon). The antibody for LMO3 (Abcam) was used to incubate the slides at 4 °C overnight with optimal dilution. HRP (rabbit) second antibody (Huabio) was used to incubate above slides at room temperature for 1 h. The slides were labeled with DAB substrate liquid (Thermo Scientific) and counterstained by hematoxylin. All the sections were photographed with a microscope (Carl Zeiss). Scoring was designated according to the ratio and intensity of positive-staining cells: 0–5% scored 0; 6–40% scored 1; 41–70% scored 2; more than 70% scored 3. The final score was defined as low or high expression group as follows: score 0–1, low expression, score 2–3, high expression. All scores were determined independently by more than two senior pathologists in a blinded manner.

### Western blotting and GTPase pull-down assays

Cells were lysed in lysis buffer. Then the proteins were separated by SDS-PAGE under reducing condition. The membranes were blocked in phosphate-buffered saline/Tween-20 containing 5% BSA, then incubated by the antibodies for LMO3 (Abcam), phospho-YAP (Cell Signaling), total-YAP (Cell Signaling), phospho-LATS1 (Cell Signaling), total-LATS1 (Cell Signaling), GAPDH (Huabio) and species-specific secondary antibodies separately. The membranes were detected by Odyssey imaging system (LI-COR). GTPase pull-down assays were performed according to standard procedures as described [18].

### siRNA or shRNA transfection

Small interfering RNAs duplexes for LMO3 used in this study was produced by Genepharma. Transfection steps were performed according to the manufacture’s protocols. The sequences of siRNA were designed as: si-LMO3–1: F: GGACUACGAGGAAGGUUUAdTdT, R: UAAACCUUCCUCGUAGUCCdTdT; si-LMO3–2: F: GCUGCAACCGAAAGAUCAAdTdT, R: UUGAUCUUUCGGUUGCAGCdTdT. Further, shRNA sequence was designed as: sh-LMO3: F: GATCCGTACACTAAAGCTAATCTT ATCTTCCTGTCAGAATAAGATTAGCTTTAGTGTACTTTTTG, R: AATTCAAAAAGTACACTAAAGCTAATCTTATTCTGACAGGAAGATAAGATTAGCTTTAGTGTACG. The structure of pGreenPuro used for shRNA and vector construction was shown in Additional file 2: Figure S2.

### rLMO3 protein and inhibitors

Recombinant LMO3 (rLMO3) protein was purchased from Abnova. The inhibitor of Hippo (Verteporfin and Peptide 17) were purchased from Selleck.

### In vitro invasion assay

MHCC-97H or SMMC-7721 cells were detached by 0.25% trypsin/0.01% EDTA in 1 × PBS and resuspended in serum-free DMEM medium. 2 × 10^4^ cells in 100 μl were added into matrigel (BD)-coated inserts (Millipore) seated on the 24-well plate. Then DMEM medium contained 5% FBS was added into the bottom chamber. After the cells were incubated at 37 °C for 48 h, filters were fixed and stained with 0.1% (*w*/*v*) Crystal Violet. Non-invading cells were removed firstly, and invading cells were counted under a microscope at a magnification of 400×. About 3 grids per field were counted. All of the experiments were repeated twice.

### Anoikis assays

5 × 10^5^ MHCC-97H or SMMC-7721 cells were cultured on poly-HEMA treated 12-well plates at 37 °C for 48 h. Then the adherent cells were detached and harvested in complete DMEM medium and centrifuged at 1000 rpm/5 min. The cells were washed with 1 × PBS and incubated with 100 μl binding buffer containing 3.5 μl Annexin V and 3.5 μl propidium iodide (PI) at room temperature for 15 min. All of the cells were analyzed by flow cytometry (BD).

### Edu assay

1 × 10^6^ MHCC-97H or SMMC-7721 cells were seeded into 6-well plates. 50 μM of Edu from Edu Apollo^®^ 488 In Vitro Flow Cytometry Kit (RiBoBio) was added into the plates 2 h before harvesting the cells. Cells were collected and centrifuged at 1000 rpm/5 min, and supernatant was removed. For fixation, 4% paraformaldehyde was added into the cells and incubated for 15 min, and washed once by 1 × PBS. Then cells were resuspended in Tris buffer saline with 0.5% Triton X-100 and incubated for 10 min, and washed again with 1 × PBS. Amounts of 500 μl staining solution with Apollo^®^ 488 fluorescent azide was added into cells, incubated for 10 min, and then rinsed twice with Tris buffer saline with 0.5% Triton X-100. All of the cells were analyzed by flow cytometry (BD).

### In vivo metastasis assays

2 × 10^6^ SMMC-7721 cells infected with sh-LMO3 or control, were detached and suspended in 30 μl serum-free DMEM/matrigel (1:1) for each BALB/c-nu/nu mouse. Through a 1 cm transverse incision in the upper abdomen under anesthesia, each mouse (6 weeks, male, 10 in each group) was orthotopically inoculated in the left hepatic lobe with a microsyringe. Meanwhile, 1 × 10^6^ SMMC-7721 cells infected with sh-LMO3 or control were injected intravenously into nude mice (6 in each group). Mice were sacrificed after 6 weeks. The livers and lungs were dissected, fixed with phosphate-buffered neutral formalin and prepared for standard histological examination. All of mice were manipulated and housed according to protocols approved by the Shanghai University of Traditional Chinese Medicine Animal Care Commission. All animals received humane care according to the criteria outlined in the “Guide for the Care and Use of Laboratory Animals” prepared by the National Academy of Sciences and published by the National Institutes of Health.

### Co-immunoprecipitation

For intracellular immunoprecipitation, Huh-7 cell lysates transfected with HA-tagged LMO3 or vector control were subjected to immunoprecipitation with anti-HA monoclonal antibody (Millipore) or control IgG. Then the immunoblotting with anti-LATS1 or YAP antibodies was performed.

### Statistical analysis

Values are expressed as the mean ± standard error of the mean. Statistical analyses were performed using SPSS 16.0 for windows. Survival time was analyzed with the Kaplan-Meier method. The association between LMO3 expression and the clinicopathological features of HCC patients was evaluated using Pearson’s Chi-square test. One-way analysis of variance was used for comparison between groups. *P* < 0.05 was considered to indicate a statistically significant difference.

## Results

### LMO3 is upregulated in HCC, and closely related with the clinicopathological findings and patient prognoses

**Fig. 1:**
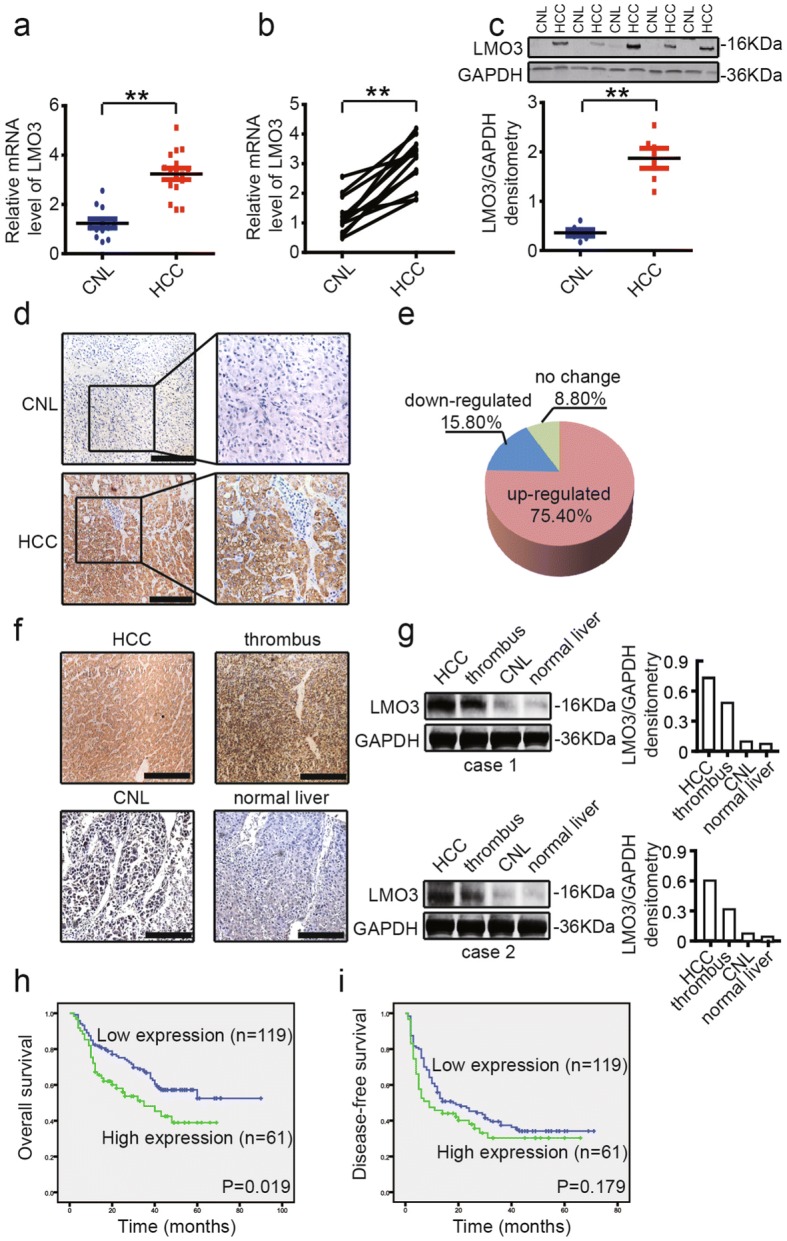
LMO3 expression is upregulated in HCC and closely related with clinicopathological findings or patient prognoses. **a** The mRNA expression level of LMO3 in 16 cases HCC and 12 cases corresponding noncancerous liver (CNL) tissues. **b** The mRNA expression level of LMO3 in 12 paired HCC and CNL tissues. **c** The protein expression level of LMO3 in 5 paired HCC and CNL tissues. **P < 0.01. **d** The immunohistochemical staining of LMO3 in HCC and CNL tissues. Scale bars: 10 μm. **e** By analyzing HCC tissue microarray (*n* = 180), the expression of LMO3 was found to be upregulated in 75.40% HCC tissues. **f** The immunohistochemical staining of LMO3 in HCC, thrombus, CNL and normal liver tissues. Scale bars: 5 μm. **g** Western blotting analysis of LMO3 expression in HCC, thrombus, CNL and normal liver tissues. GAPDH was used as a loading control. **h** and **i** Kaplan-Meier analysis of overall survival (OS) (**f**, *P* = 0.019) and disease-free survival (DFS) (**g**, *P* = 0.179) for the expression of LMO3

To investigate the expression level of LMO3 in HCC tissues, we collected 16 cases HCC and 12 cases CNL tissues. Through Quantitative real-time PCR, we found that the expression of LMO3 was significantly upregulated in HCC tissues (Fig. 1a). In 12 paired HCC and CNL tissues, the mRNA expression of LMO3 was found to be significantly upregulated in HCC tissues (Fig. 1b). Furthermore, in 5 paired HCC and CNL tissues, the protein expression of LMO3 was also found to be significantly upregulated in HCC tissues (Fig. 1c).

Then we used an HCC tissue microarray (*n* = 180) to detect the correlation between LMO3 expression and clinicopathological findings and patient prognoses (Additional file 3: Table S2). We found that the expression of LMO3 was upregulated in 75.40% HCC tissues (Fig. 1e). The upregulated LMO3 expression in HCC tissues was further confirmed by immunohistochemical staining (Fig. 1d). Also, we found that the expression of LMO3 was closely related to patient tumor encapsulation, thrombus, vascular invasion and TNM stage (Table 1). To investigate the relevance of LMO3 with HCC metastasis, we collected HCC, tumor thrombus, CNL and normal liver tissues from the same patients and detected LMO3 expression in these tissues. Through immunohistochemical staining and western blotting, we found that LMO3 expression was higher in the HCC and tumor thrombus tissues, and lower in the CNL and normal liver tissues (Fig. 1f, g). Additionally, we found that the high expression of LMO3 was positively correlated with poor overall patient survival (OS) (*P* = 0.019), while it was not correlated with patient disease-free survival (DFS) (*P* = 0.179) (Fig. 1h, i).

**Table 1:** Correlation of the clinicopathological findings with LMO3 expression

Variable		LMO3 (n)		
		High	Low	*P*
Age	≤50 years	28	65	0.382
	> 50 years	35	62	
Gender	Female	7	20	0.389
	Male	56	107	
Hepatitis history	Yes	53	116	0.263
	No	10	11	
Gamma-glutamytransferase	≤50(U/L)	22	50	0.605
	> 50(U/L)	40	77	
Alpha-fetoprotein	≤20 ng/mL	18	49	0.185
	> 20 ng/mL	44	77	
Glu	≤7 mmol/L	6	11	0.793
	> 7 mmol/L	55	116	
Tumor multiplicity	Single	49	111	0.087
	Multiple	14	16	
Tumor satelite	Yes	19	39	0.938
	No	44	88	
*Tumor encapsulation	**Incomplete**	**26**	**34**	**0.029**
	**Complete**	**35**	**93**	
*Tumor thrombus	**Yes**	**22**	**18**	**< 0.001**
	**No**	**41**	**109**	
Tumor differentiation	I	2	1	0.458
	II	23	49	
	III	38	76	
*Vascular invasion	**Yes**	**24**	**27**	**0.014**
	**No**	**39**	**100**	
Tumor size	≤5 cm	29	71	0.199
	> 5 cm	34	56	
*TNM stage	**I**	**31**	**86**	**0.020**
	**II**	**7**	**15**	
	**III**	**25**	**25**	

Pearson’s χ^2^ test was used. The bolding stands for the *P*-values with significant differences

### Knockdown of LMO3 suppresses the invasion and anoikis inhibition of HCC cells in vitro

**Fig. 2:**
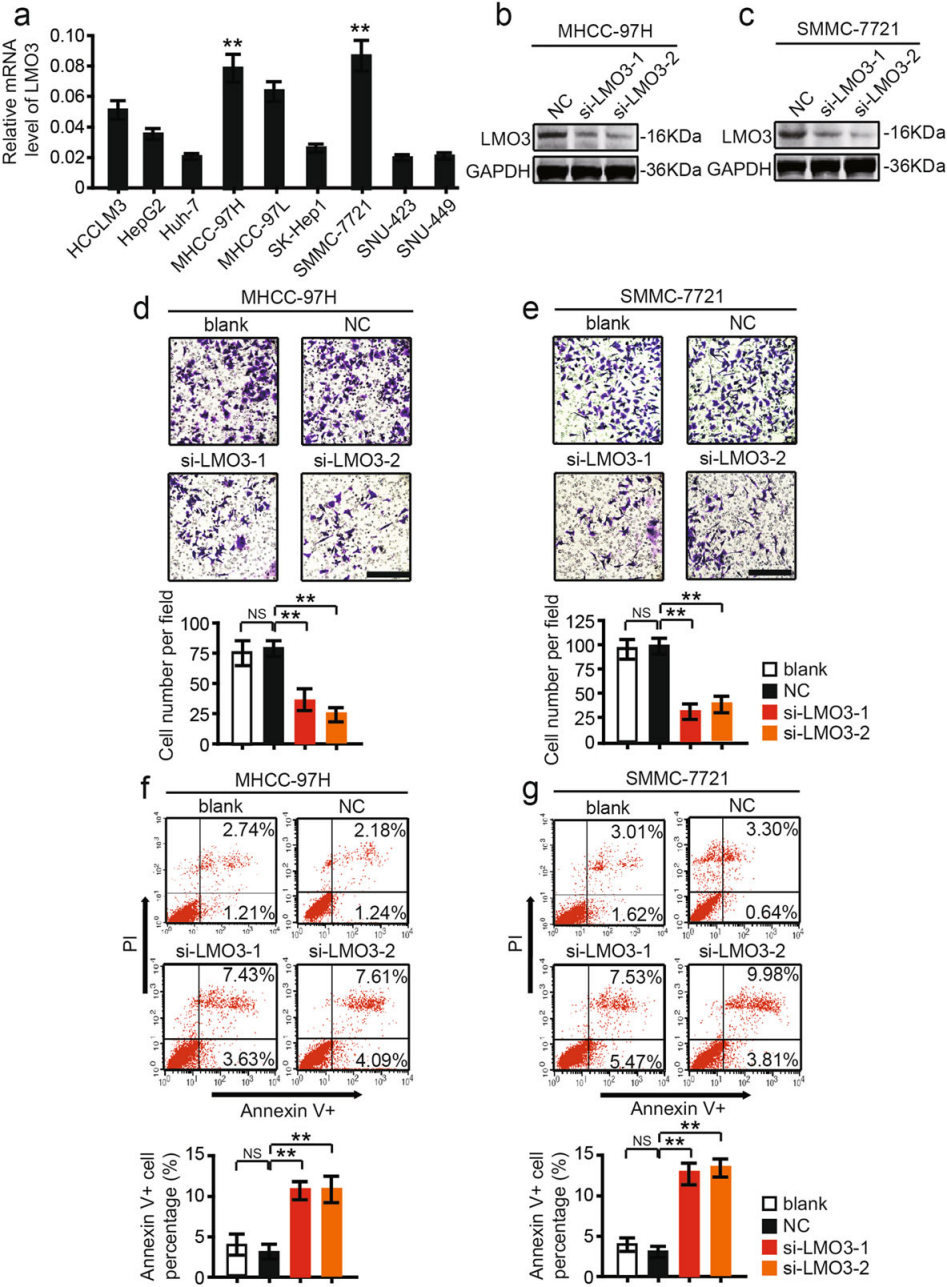
LMO3 knockdown suppresses the invasion and anoikis inhibition of HCC cells in vitro. **a** Expression of LMO3 in HCC cell lines, including HCCLM3, HepG2, Huh-7, MHCC-97H, MHCC-97 L, SK-Hep1, SMMC-7721, SNU-423 and SNU-449 cells. **b** and **c** The protein expression level of LMO3 in MHCC-97H (**b**) and SMMC-7721 (**c**) cells, which were infected with siRNA of LMO3 or NC. **d** Representative photos of invaded MHCC-97H cells infected with siRNA of LMO3 or NC. Statistical analysis of invaded MHCC-97H cells in the three groups is shown below. Scale bars: 10 μm. **e** Representative photos of invaded SMMC-7721 cells infected with siRNA of LMO3 or NC. Statistical analysis of invaded SMMC-7721 cells in the three groups is shown below. Scale bars: 10 μm. **f** Flow cytometry analysis of anoikis of MHCC-97H cells infected with siRNA of LMO3 or NC. Flow cytometry statistical analysis of apoptotic MHCC-97H cells in the two groups is shown below. **g** Flow cytometry analysis of anoikis of SMMC-7721 cells infected with siRNA of LMO3 or NC. Flow cytometry statistical analysis of apoptotic SMMC-7721 cells in the two groups is shown below. **P < 0.01, NS, no significant

To investigate the biological functions of LMO3 in HCC, we detected the expression level of LMO3 in 9 HCC cell lines. As shown in Fig. 2a, we found that LMO3 had high expression levels in MHCC-97H and SMMC-7721 cells. Then we selected MHCC-97H and SMMC-7721 cells and knocked down LMO3 by using siRNA (labeled as si-LMO3–1 and si-LMO3–2). Through western blotting analyses, we found that LMO3 was successfully silenced in MHCC-97H (Fig. 2b) and SMMC-7721 cells (Fig. 2c).

Then we investigated the role of LMO3 in the invasion of HCC cells. By transwell matrigel invasion assay, it was found that knockdown of LMO3 suppressed the invasiveness of MHCC-97H and SMMC-7721 (Fig. 2d, e) cells after 48 h. Furthermore, through annexin V anoikis assay, we found that the knockdown of LMO3 promoted the anoikis of MHCC-97H and SMMC-7721 (Fig. 2f, g) cells after 48 h. Additionally, by Edu assay, we found that the proliferation of MHCC-97H and SMMC-7721 cells was not affected by LMO3 knockdown at 24-, 48- and 72-h time points respectively (Additional file 4: Figure S1a, b).

### Knockdown of LMO3 suppresses the intrahepatic and distant metastasis of HCC in vivo

**Fig. 3:**
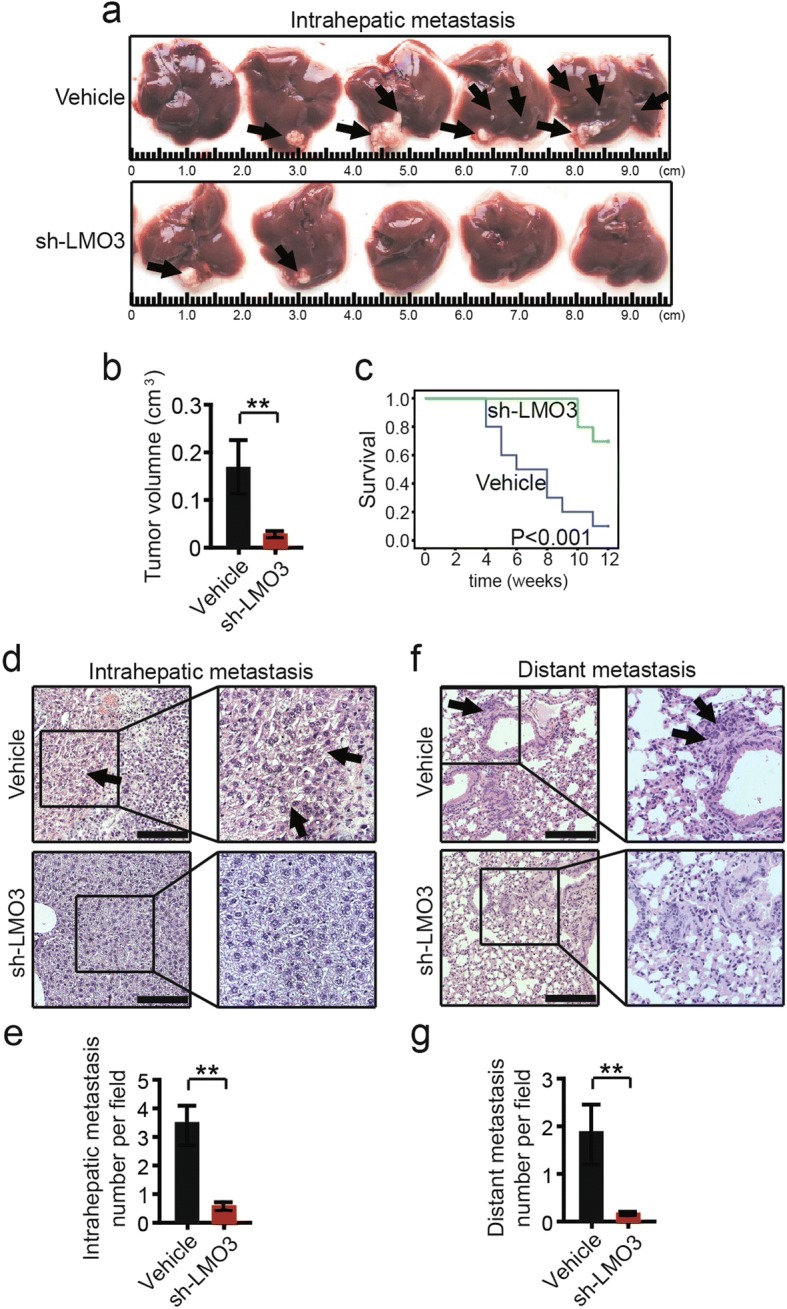
LMO3 knockdown suppresses the metastasis of HCC cells in vivo. **a** Representative photos of liver tissues from mice orthotopically inoculated with LMO3 silenced SMMC-7721 and control cells (*n* = 5 each group). **b** Statistical analysis of intrahepatic metastatic tumor volume is shown right. **c** Mice survival of LMO3 silenced SMMC-7721 and control group (*n* = 10 each group) (*P* < 0.001). **d** Representative images of H&E staining in liver tissues from mice orthotopically inoculated with LMO3 silenced SMMC-7721 and control cells (*n* = 5 each group). Scale bars: 10 μm. **e** Statistical analysis of intrahepatic metastatic focis in the two groups is shown below. **f** Pulmonary metastases were detected by H&E staining (*n* = 6 each group). Scale bars: 10 μm. **g** Statistical analysis of numbers of pulmonary metastatic nodules is shown below. **P < 0.01

To further investigate the role of LMO3 in cancer progression in vivo, sh-LMO3 and control cells (SMMC-7721) were orthotopically injected into nude mice to assess intrahepatic metastases and intravenously transplanted to assess distant metastases. After 6 weeks, we found that the intrahepatic metastatic nodules in the sh-LMO3 group were obviously less than in the vehicle group (Fig. 3a, b). Mice survival in the LMO3-silenced group was significantly increased compared to the vehicle group (Fig. 3c). Histological staining of the liver tissues indicated that mice transplanted with sh-LMO3 cells had fewer intrahepatic metastatic nodules than those transplanted with control cells (Fig. 3d, e). Meanwhile, the pulmonary metastatic nodules in mice transplanted with sh-LMO3 cells were less than those transplanted with control cells (Fig. 3f, g).

### LMO3 knockdown increases the phosphorylation of YAP/LATS1 signaling and decreases Rho GTPases activities

**Fig. 4:**
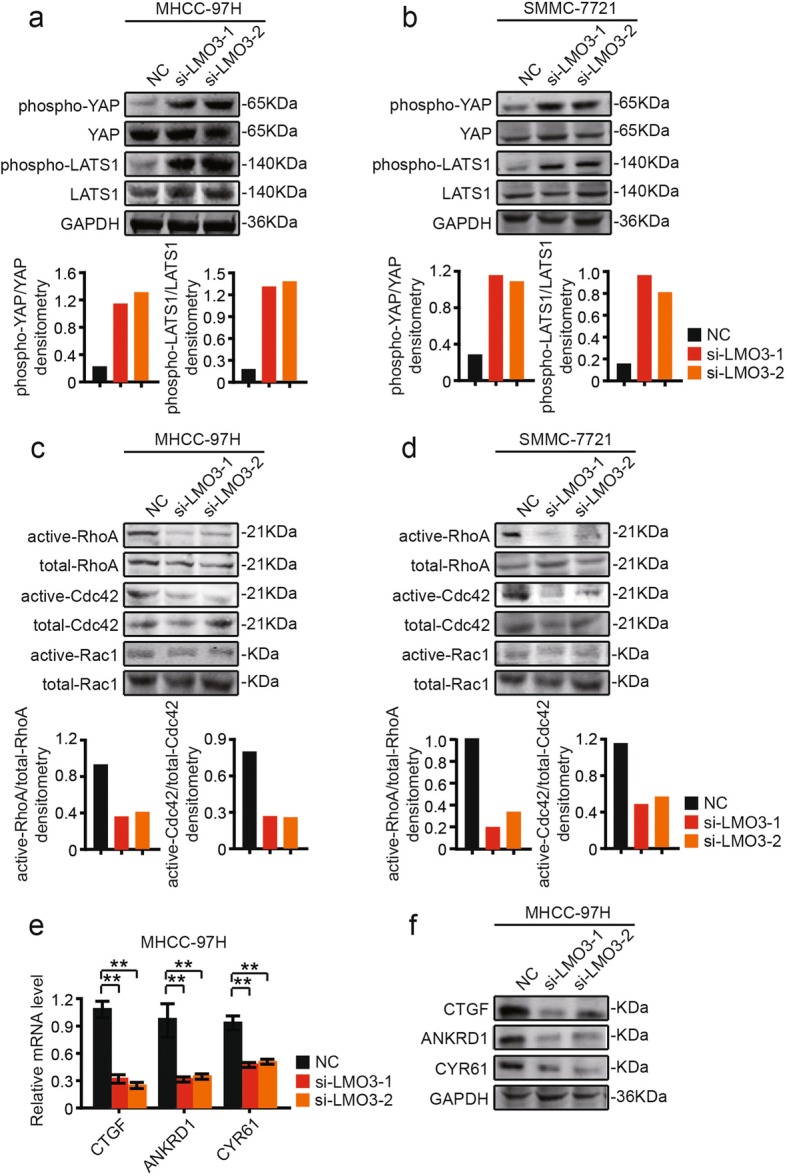
Knockdown of LMO3 increases the phosphorylation of YAP/LATS1 and decreases Rho GTPases activities. **a** and **b** Western blotting analysis of phospho-YAP, YAP, phospho-LATS1 and LATS1 in LMO3 knockdown and control MHCC-97H (a) or SMMC-7721 cells (b). Statistical analyses of phospho-YAP/YAP and phospho-LATS1/LATS1 densitometry are shown below. **c** and **d** MHCC-97H (c) or SMMC-7721 (d) cells were serum starved for 24 h and the activities of RhoA, Cdc42 and Rac1 were measured by pull-down assays in LMO3 knockdown and control cells. Statistical analyses of active-RhoA/total-RhoA and active-Cdc42/total-Cdc42 densitometry are shown below. **e** The mRNA levels of CTGF, ANKRD1 and CYR61 in LMO3 knockdown and control MHCC-97H cells. **P < 0.01. **f** Western blotting analysis of CTGF, ANKRD1 and CYR61 in LMO3 knockdown and control MHCC-97H cells

We further investigated the underlying mechanism of LMO3 in HCC. Through western blotting analyses, we found that the Hippo signaling pathway played important roles in HCC invasion and metastasis. Interestingly, knockdown of LMO3 in MHCC-97H or SMMC-7721 cells significantly increased the phosphorylation of YAP. Furthermore, the phosphorylation of LATS1, a core component of the Hippo pathway, was also increased by silencing LMO3 (Fig. 4a, b). Then LMO3 knockdown and control MHCC-97H or SMMC-7721 cells were serum starved for 24 h. The activities of RhoA and Cdc42 were measured by pull-down assays. We found that RhoA and Cdc42 activities were suppressed by LMO3 knockdown. Rac1 activity had no obvious change in LMO3 knockdown and control MHCC-97H or SMMC-7721 cells (Fig. 4c, d).

We also detected the mRNA levels of canonical YAP target genes, including Connective Tissue Growth Factor (CTGF), Ankyrin Repeat Domain 1 (ANKRD1), and Cysteine Rich Angiogenic Inducer 61 (CYR61). It was found that CTGF, ANKRD1 and CYR61 mRNA or protein levels were significantly suppressed in LMO3-silenced MHCC-97H cells (Fig. 4e). These results suggested that the Hippo pathway was activated in LMO3-silenced HCC cells.

### LMO3 directly interacts with LATS1, and recombinant LMO3 protein administration decreases the phosphorylation of YAP/LATS1 signaling and increases Rho GTPases activities

**Fig. 5:**
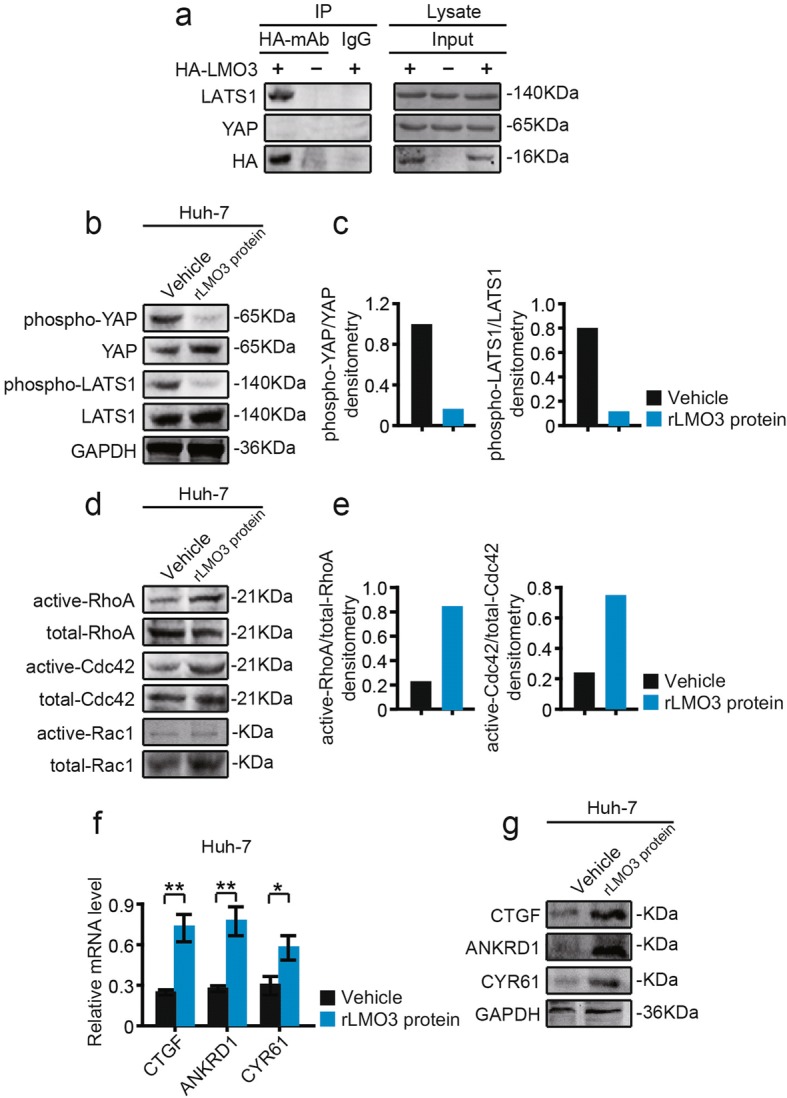
LMO3 directly interacts with LATS1, and recombinant LMO3 protein administration suppresses the phosphorylation of YAP/LATS1 and increases Rho GTPases activities. **a** Co-immunoprecipitation of LMO3 with LATS1 or YAP. Huh-7 cell lysates transfected with HA-tagged LMO3 or vector control were subjected to immunoprecipitation with anti-HA monoclonal antibody or control IgG, followed by immunoblotting with anti-LATS1 or YAP antibodies. The input control on the right panel shows the levels of transfected HA-LMO3 and LATS1 or YAP in HA-tagged LMO3 or vector control transfected cells. **b** and **c** Western blotting analysis of phospho-YAP, YAP, phospho-LATS1 and LATS1 in recombinant LMO3 (rLMO3) protein treated and control Huh-7 cells (**b**). Statistical analyses of phospho-YAP/YAP and phospho-LATS1/LATS1 densitometry are shown right (**c**). **d** and **e** Huh-7 cells were serum starved for 24 h and the activities of RhoA and Cdc42 were measured by pull-down assays in rLMO3 protein treated and control cells (**d**). Statistical analyses of active-RhoA/total-RhoA and active-Cdc42/total-Cdc42 densitometry are shown right (**e**). **f** The mRNA levels of CTGF, ANKRD1 and CYR61 in rLMO3 protein treated and control Huh-7 cells. *P < 0.05, **P < 0.01. **g** Western blotting analysis of CTGF, ANKRD1 and CYR61 in rLMO3 protein treated and control Huh-7 cells

Co-immunoprecipitation experiments were performed to investigate whether LMO3 was directly associated with the components of the Hippo pathway. First, Huh-7 was transfected with HA-tagged LMO3 or vector control. We found that immunoprecipitates of LMO3 from Huh-7 cells contained LATS1, but no YAP (Fig. 5a). Furthermore, we added recombinant LMO3 (rLMO3) protein into Huh-7 cells, in which LMO3 had a low expression level. The results showed that the phosphorylation of YAP or LATS1 was significantly suppressed after treatment with rLMO3 protein (Fig. 5b, c). Additionally, RhoA and Cdc42 activities were increased by rLMO3 protein administration, while Rac1 activity had no obvious change (Fig. 5d, e).

Meanwhile, we also found that CTGF, ANKRD1 and CYR61 mRNA or protein levels were significantly increased in rLMO3 protein treated Huh-7 cells (Fig. 5f). Thus, we conclude that LMO3 interacts with LATS1, increases Rho GTPases activities and suppresses the Hippo signaling pathway.

### Hippo pathway inhibitors could abrogate recombinant LMO3 protein-induced HCC cell invasion and anoikis inhibiton

**Fig. 6:**
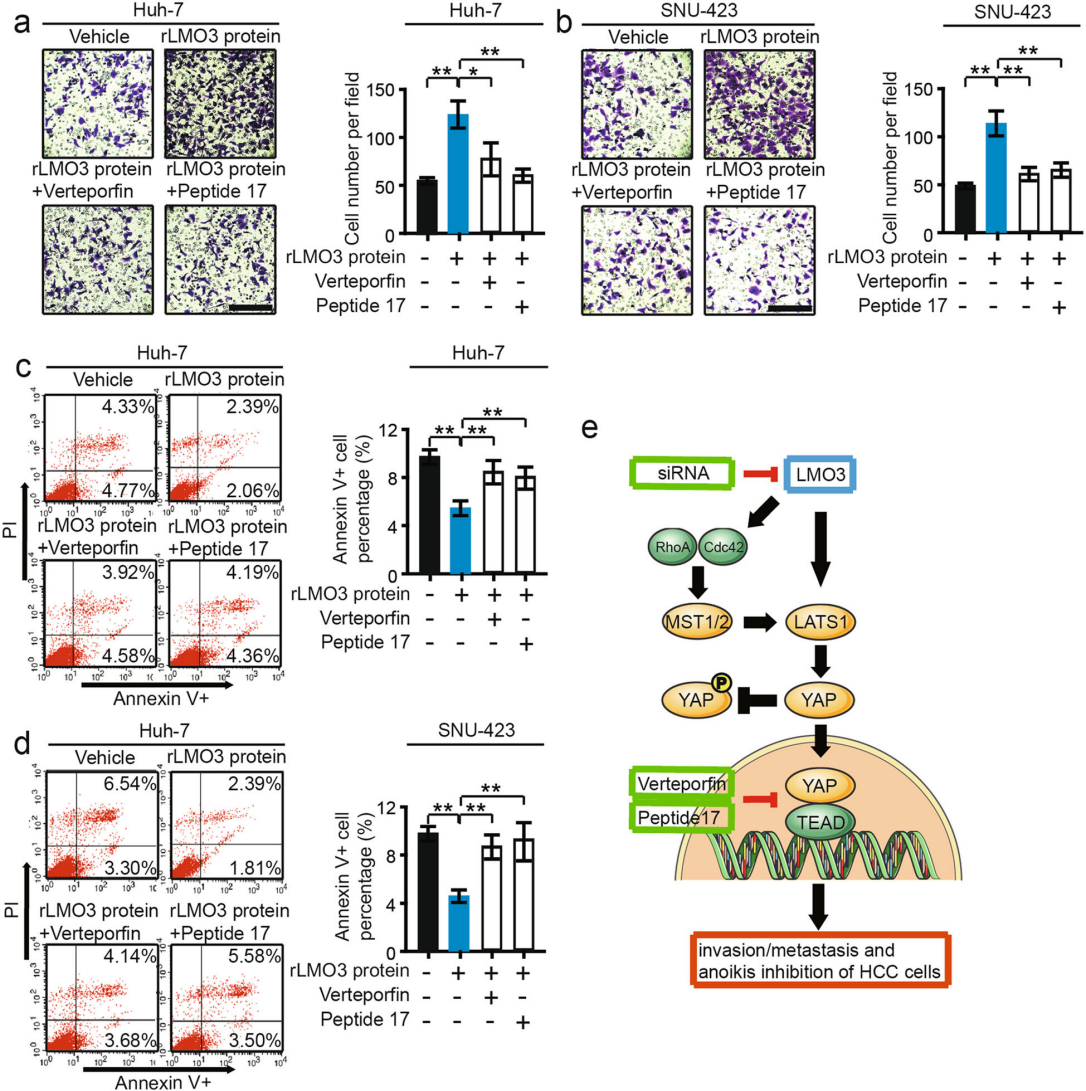
Hippo pathway inhibitors abrogate recombinant LMO3 protein induced HCC cell invasion and anoikis inhibiton. **a** and **b** Huh-7 and SNU-423 cells were treated with 50 nM rLMO3 protein, 50 nM rLMO3 protein plus 50 nM Verteporfin (the inhibitor of YAP-TEAD), 50 nM rLMO3 protein plus 50 nM Peptide 17 (the inhibitor of YAP-TEAD) respectively. The invaded Huh-7 (**a**) and SNU-423 (**b**) cells were analyzed after 48 h. Scale bars: 10 μm. **c** and d Huh-7 and SNU-423 cells were treated with 50 nM rLMO3 protein, 50 nM rLMO3 protein plus 50 nM Verteporfin, 50 nM rLMO3 protein plus 50 nM Peptide 17 respectively. The apoptotic Huh-7 (**c**) and SNU-423 (**d**) cells were analyzed after 48 h. *P < 0.05, **P < 0.01. **e** LMO3 promoted HCC cell invasion and anoikis inhibition by interacting with LATS1 and suppressing Hippo signaling

By using Verteporfin (the inhibitor of YAP-TEAD) and Peptide 17 (YAP-TEAD inhibitor I, the inhibitor of YAP-TEAD), we further investigated the effects of LMO3 on HCC cell invasion and anoikis inhibition. rLMO3 protein was added into Huh-7 and SNU-423 cells, in which LMO3 had low expression levels. Then, Verteporfin and Peptide 17 were added after 2 h. We found that Verteporfin and Peptide 17 abrogated rLMO3 protein-induced Huh-7 or SNU-423 cell invasion (Fig. 6a, b). Meanwhile, the anoikis inhibition of Huh-7 or SNU-423 cells induced by rLMO3 protein was also abrogated by these inhibitors (Fig. 6c, d).

These results indicate that LMO3 promoted HCC cell invasion and anoikis inhibition by interacting with LATS1 and suppressing Hippo signaling (Fig. 6e).

## Discussion

In recent years, studies on LMO3 in some types of cancers have been reported [14–17]. Nevertheless, the detailed biological functions and related mechanism of LMO3 in HCC were first investigated in this research. We found that LMO3 expression was closely related with tumor metastasis related clinicopathological findings and patient prognoses. Our experiments in vitro and in vivo revealed that knockdown of LMO3 suppresses cell invasion and anoikis inhibition in HCC. All of the above data suggested that LMO3 is involved in the invasion, metastasis and anoikis inhibition of HCC cells.

Invasion and metastasis are major concerns during the progression of cancer [19–23]. The Hippo pathway is known to be pivotal in modulating the invasion and metastasis of tumor cells [24–27]. YAP is known to contribute to metastasis via multiple mechanisms. YAP interacts with TEAD and FOS in the nucleus, and reprograms gene expression to induce epithelial-mesenchymal transition (EMT). YAP also antagonizes E-cadherin junction assembly by regulating actin cytoskeleton organization and contributes to EMT. Furthermore, YAP activation supports stiffening of the extracellular matrix of cancer-associated fibroblasts (CAFs) to enhance YAP nuclear localization in tumor cells. Such interplay between cancer cells and CAFs might amplify the effects of YAP during tumorigenesis [28–31].

LMO3 has been proved to promote cell invasion or proliferation through Akt-mTOR/GSK3β signaling in gastric cancer [32]. In this research, we found that knockdown of LMO3 increased the phosphorylation of YAP and LATS1, and thus restrained them in the cytoplasm where they lost their transcriptional activation. Furthermore, these results were confirmed by detecting Rho GTPases activities and canonical YAP target genes. Meanwhile, administration of rLMO3 protein led to an opposite effect of LMO3 in the Hippo pathway.

The studies of Aoyama et al. and Isogai et al. showed that LMO3 interacts with HEN2 and enhances cell growth in neuroblastoma [14, 15]. Interestingly, in this research we found that LMO3 directly interacts with LATS1, and thus it suppressed Hippo signaling. Identifying the interaction between LMO3 and LATS1 provides direct evidence for the important role of LMO3 in the regulation of Hippo signaling. Also, we found that rLMO3 protein-induced HCC cell invasion and anoikis inhibition is abrogated by the inhibitors of the Hippo pathway, indicating that LMO3-induced HCC cell invasion, metastasis and anoikis inhibition are dependent on the suppression of Hippo signaling.

## Conclusions

In conclusion, we found that LMO3 plays an important role in HCC cell invasion, metastasis and anoikis inhibition. High expression levels of LMO3 in HCC suppresses the Hippo signaling pathway by interacting with LATS1, and thus LMO3 promotes the invasion and metastasis of HCC cells. LMO3 may be used as a potential therapeutic strategy for HCC in future.

## Additional files

Additional file 1: **Table S1** Primer sequences used for human LMO3, CTGF, ANKRD1 and CYR61 detection. **a** Edu assay of MHCC-97H cells infected with siRNA of LMO3 at 0, 24, 48 and 72 h time points respectively. **b** Edu assay of SMMC-7721 cells infected with siRNA of LMO3 at 0, 24, 48 and 72 h time points respectively. **P < 0.01. (DOC 30 kb)

Additional file 2: **Figure S2** The structure of pGreenPuro used for shRNA and vector construction. (PDF 622 kb)

Additional file 3: **Table S2** The original data of tissue microarray assay. (DOC 468 kb)

Additional file 4: **Figure S1** LMO3 knockdown has no effects on the proliferation of HCC cells. Multiplicity: 1 – single, 2 - multiple; Satelite: 0 – no, 1 or 2 - yes; Encapsulation: 0 – complete, 1 - incomplete; Vascular invasion: 0 – no, 1 - yes; Tumor thrombus: 0 – no, 1 - yes. (PDF 505 kb)

## Abbreviations

ANKRD1: Ankyrin Repeat Domain 1
CNL: Corresponding non-cancerous liver
CTGF: Connective Tissue Growth Factor
CYR61: Cysteine Rich Angiogenic Inducer 61
DFS: Disease-free survival
HCC: Hepatocellular carcinoma
LATS1: Large Tumor Suppressor Kinase 1
LMO3: LIM domain only 3
OS: Overall survival
rLMO3: recombinant LMO3
YAP: Yes Associated Protein

## References

[1] El-Serag HB, Rudolph KL (2007). Hepatocellular carcinoma: epidemiology and molecular carcinogenesis. Gastroenterology.

[2] Gish RG, Porta C, Lazar L, Ruff P, Feld R, Croitoru A (2007). Phase III randomized controlled trial comparing the survival of patients with unresectable hepatocellular carcinoma treated with nolatrexed or doxorubicin. J Clin Oncol.

[3] Aravalli RN, Steer CJ, Cressman EN (2008). Molecular mechanisms of hepatocellular carcinoma. Hepatology.

[4] Bacac M, Stamenkovic I (2008). Metastatic cancer cell. Annu Rev Pathol.

[5] Zender L, Villanueva A, Tovar V, Sia D, Chiang DY, Llovet JM (2010). Cancer gene discovery in hepatocellular carcinoma. J Hepatol.

[6] Mínguez B, Hoshida Y, Villanueva A, Toffanin S, Cabellos L, Thung S (2011). Gene-expression signature of vascular invasion in hepatocellular carcinoma. J Hepatol.

[7] Yimlamai D, Fowl BH, Camargo FD (2015). Emerging evidence on the role of the Hippo/YAP pathway in liver physiology and cancer. J Hepatol.

[8] Wu H, Wei L, Fan F, Ji S, Zhang S, Geng J (2015). Integration of Hippo signalling and the unfolded protein response to restrain liver overgrowth and tumorigenesis. Nat Commun.

[9] Janse van Rensburg HJ, Yang X (2016). The roles of the Hippo pathway in cancer metastasis. Cell Signal.

[10] Juan Wen, Hong Wanjin (2016). Targeting the Hippo Signaling Pathway for Tissue Regeneration and Cancer Therapy. Genes.

[11] Rabbitts TH (1998). LMO T-cell translocation oncogenes typify genes activated by chromosomal translocations that alter transcription and developmental processes. Genes Dev.

[12] Sum EY, Segara D, Duscio B, Bath ML, Field AS, Sutherland RL (2005). Overexpression of LMO4 induces mammary hyperplasia, promotes cell invasion, and is a predictor of poor outcome in breast cancer. Proc Natl Acad Sci U S A.

[13] Visvader JE, Venter D, Hahm K, Santamaria M, Sum EY, O’Reilly L (2001). The LIM domain gene LMO4 inhibits differentiation of mammary epithelial cells in vitro and is overexpressed in breast cancer. Proc Natl Acad Sci U S A.

[14] Aoyama M, Ozaki T, Inuzuka H, Tomotsune D, Hirato J, Okamoto Y (2005). LMO3 interacts with neuronal transcription factor, HEN2, and acts as an oncogene in neuroblastoma. Cancer Res.

[15] Isogai E, Ohira M, Ozaki T, Oba S, Nakamura Y, Nakagawara A (2011). Oncogenic LMO3 collaborates with HEN2 to enhance neuroblastoma cell growth through transactivation of Mash1. PLoS One.

[16] Song YF, Hong JF, Liu DL, Lin QA, Lan XP, Lai GX (2015). miR-630 targets LMO3 to regulate cell growth and metastasis in lung cancer. Am J Transl Res.

[17] Watanabe H, Francis JM, Woo MS, Etemad B, Lin W, Fries DF (2013). Integrated cistromic and expression analysis of amplified NKX2-1 in lung adenocarcinoma identifies LMO3 as a functional transcriptional target. Genes Dev.

[18] Zhang Z (2011). Migration of epithelial cells on laminins: RhoA antagonizes directionally persistent migration. Eur J Cell Biol.

[19] Liu Y, Ding Y, Huang J, Wang S, Ni W, Guan J (2014). MiR-141 suppresses the migration and invasion of HCC cells by targeting Tiam1. PLoS One.

[20] Wang YH, Dong YY, Wang WM, Xie XY, Wang ZM, Chen RX (2013). Vascular endothelial cells facilitated HCC invasion and metastasis through the Akt and NF-κB pathways induced by paracrine cytokines. J Exp Clin Cancer Res.

[21] Chen B, Tang J, Guo YS, Li Y, Chen ZN, Jiang JL (2013). Calpains are required for invasive and metastatic potentials of human HCC cells. Cell Biol Int.

[22] Xie B, Xing R, Chen P, Gou Y, Li S, Xiao J (2010). Down-regulation of c-Met expression inhibits human HCC cells growth and invasion by RNA interference. J Surg Res.

[23] Cui JF, Liu YK, Zhang LJ, Shen HL, Song HY, Dai Z (2006). Identification of metastasis candidate proteins among HCC cell lines by comparative proteome and biological function analysis of S100A4 in metastasis in vitro. Proteomics.

[24] Han Q, Lin X, Zhang X, Jiang G, Zhang Y, Miao Y (2017). WWC3 regulates the Wnt and Hippo pathways via Dishevelled proteins and large tumour suppressor 1, to suppress lung cancer invasion and metastasis. J Pathol.

[25] Tong R, Yang B, Xiao H, Peng C, Hu W, Weng X (2017). KCTD11 inhibits growth and metastasis of hepatocellular carcinoma through activating Hippo signaling. Oncotarget.

[26] Varzavand A, Hacker W, Ma D, Gibson-Corley K, Hawayek M, Tayh OJ (2016). α3β1 Integrin Suppresses Prostate Cancer Metastasis via Regulation of the Hippo Pathway. Cancer Res.

[27] Wei Changran, Wang Ying, Li Xiangqi (2018). The role of Hippo signal pathway in breast cancer metastasis. OncoTargets and Therapy.

[28] Salcedo Allende MT, Zeron-Medina J, Hernandez J, Macarulla T, Balsells J, Merino X (2017). Overexpression of Yes Associated Protein 1, an Independent Prognostic Marker in Patients With Pancreatic Ductal Adenocarcinoma, Correlated With Liver Metastasis and Poor Prognosis. Pancreas.

[29] Qiao Y, Chen J, Lim YB, Finch-Edmondson ML, Seshachalam VP, Qin L (2017). YAP Regulates Actin Dynamics through ARHGAP29 and Promotes Metastasis. Cell Rep.

[30] Li C, Wang S, Xing Z, Lin A, Liang K, Song J (2017). A ROR1-HER3-lncRNA signalling axis modulates the Hippo-YAP pathway to regulate bone metastasis. Nat Cell Biol.

[31] Nokin MJ, Durieux F, Peixoto P, Chiavarina B, Peulen O, Blomme A, et al. Methylglyoxal, a glycolysis side-product, induces Hsp90 glycation and YAP-mediated tumor growth and metastasis. Elife. 2016;19. 10.7554/eLife.19375. 10.7554/eLife.19375 PMC5081250 27759563

[32] Qiu YS, Jiang NN, Zhou Y, Yu KY, Gong HY, Liao GJ (2018). LMO3 promotes gastric cancer cell invasion and proliferation through Akt-mTOR and Akt-GSK3β signaling. Int J Mol Med.

