# Supplementary material for: LMO3 promotes hepatocellular carcinoma invasion, metastasis and anoikis inhibition by directly interacting with LATS1 and suppressing Hippo signaling
Source: J Exp Clin Cancer Res. 2018 Sep 15;37:228. doi: 10.1186/s13046-018-0903-3 (PMC6139164; doi:10.1186/s13046-018-0903-3)
Supplement: Supplementary file 1 — Table S1 Primer sequences used for human LMO3, CTGF, ANKRD1 and CYR61 detection. a Edu assay of MHCC-97H cells infected with siRNA of LMO3 at 0, 24, 48 and 72 h time points respectively. b Edu assay of SMMC-7721 cells infected with siRNA of LMO3 at 0, 24, 48 and 72 h time points respectively. **P < 0.01. (DOC 30 kb) [file 13046_2018_903_MOESM1_ESM.doc]

|  | **primer sequence-F** | **primer sequence-R** |
| --- | --- | --- |
| LMO3 | TGAGCTTACTACAGGCAGCG | GGGCTCCACCCTGTACACTA |
| CTGF | TGGAGATTTTGGGAGTACGG | CAGGCTAGAGAAGCAGAGCC |
| ANKRD1 | GTGTAGCACCAGATCCATCG | CGGTGAGACTGAACCGCTAT |
| CYR61 | CCCGTTTTGGTAGATTCTGG | GCTGGAATGCAACTTCGG |
