# Supplementary figures and images for: LMO3 promotes hepatocellular carcinoma invasion, metastasis and anoikis inhibition by directly interacting with LATS1 and suppressing Hippo signaling
Source: J Exp Clin Cancer Res. 2018 Sep 15;37:228. doi: 10.1186/s13046-018-0903-3 (PMC6139164; doi:10.1186/s13046-018-0903-3)

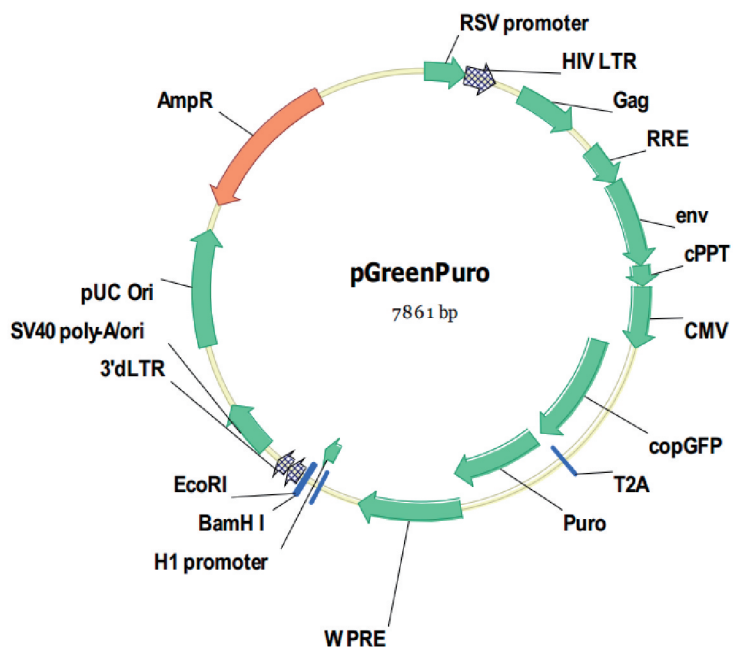

Supplementary Figure 2

Supplement: Supplementary file 2 — Figure S2 The structure of pGreenPuro used for shRNA and vector construction. (PDF 622 kb) [file 13046_2018_903_MOESM2_ESM.pdf]

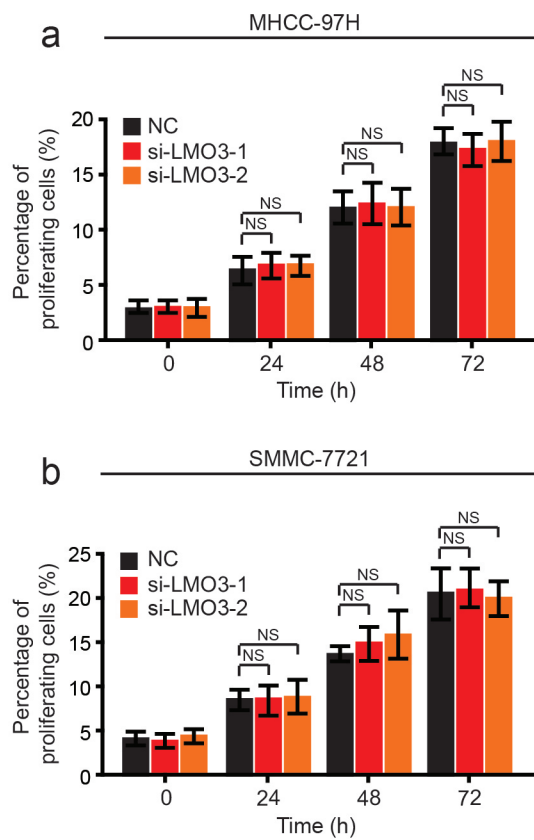

Supplementary Figure 1

Supplement: Supplementary file 4 — Figure S1 LMO3 knockdown has no effects on the proliferation of HCC cells. Multiplicity: 1 – single, 2 - multiple; Satelite: 0 – no, 1 or 2 - yes; Encapsulation: 0 – complete, 1 - incomplete; Vascular invasion: 0 – no, 1 - yes; Tumor thrombus: 0 – no, 1 - yes. (PDF 505 kb) [file 13046_2018_903_MOESM4_ESM.pdf]
