# Supplementary material for: LMO3 promotes hepatocellular carcinoma invasion, metastasis and anoikis inhibition by directly interacting with LATS1 and suppressing Hippo signaling
Source: J Exp Clin Cancer Res. 2018 Sep 15;37:228. doi: 10.1186/s13046-018-0903-3 (PMC6139164; doi:10.1186/s13046-018-0903-3)
Supplement: Supplementary file 3 — Table S2 The original data of tissue microarray assay. (DOC 468 kb) [file 13046_2018_903_MOESM3_ESM.doc]

| **NO** | **operation date** | **Sex** | **Age** | **Size (cm)** | **Multi**  **-plicity** | **Satelite** | **Encap**  **-sulation** | **Grade** | **Vascular invasion** | **Tumor thrombus** | **TNM** | **Time to reccurence** | **OS** | **Glu** | **GGT** | **AFP** |
| --- | --- | --- | --- | --- | --- | --- | --- | --- | --- | --- | --- | --- | --- | --- | --- | --- |
| 1 | 2007/9/18 | M | 27 | 14*12*9 | 1 | 0 | 0 | 3 | 1 | 1 | 3 | postoperative 1 month | postoperative 2 month | 5.1 | 391.2 | 350 |
| 2 | 2007/12/3 | M | 64 | 3.5*2.5*2; 1*1*1 | 2 | 0 | 1 | 1 | 0 | 0 | 2 | none | none | / | 26 | 3.32 |
| 3 | 2007/12/22 | F | 50 | 14*7*5 | 1 | 0 | 0 | 2 | 0 | 0 | 1 | not known | postoperative 6 month | / | 166.3 | 46.3 |
| 4 | 2008/1/16 | M | 44 | 1.5*1*1 | 1 | 0 | 1 | 2 | 0 | 0 | 1 | postoperative 30 month | none | 5.4 | 84.2 | 44 |
| 5 | 2008/6/3 | M | 43 | 15*13*9 | 1 | 0 | 0 | 2 | 1 | 1 | 4 | postoperative 2 month | postoperative 4 month | 4.3 | 195.6 | 349.6 |
| 6 | 2008/6/10 | M | 60 | 9*7*4 | 1 | 0 | 1 | 2 | 0 | 0 | 1 | postoperative 7 month | postoperative 13 month | 5.5 | 181.8 | 135.8 |
| 7 | 2008/7/16 | M | 66 | 3.5*3.5*3; 4.5*4*3 | 2 | 0 | 0 | 2 | 0 | 0 | 2 | postoperative 27 month | none | 10.1 | 171.3 | 9.5 |
| 8 | 2008/11/13 | M | 55 | 3.5*3*2.5 | 1 | 0 | 1 | 2 | 0 | 0 | 1 | postoperative 30 month | none | 4.5 | 72.3 | 4 |
| 9 | 2008/11/20 | F | 52 | 4*3*3 | 1 | 0 | 0 | 2 | 0 | 0 | 1 | postoperative 2 month | postoperative 5 month | 5.2 | 82.2 | 807.99 |
| 10 | 2008/12/22 | M | 70 | 2*2*2 | 1 | 0 | 0 | 2 | 0 | 0 | 1 | none | none | 4.8 | 16.6 | 8.1 |
| 11 | 2009/2/24 | M | 50 | 11*11*10 | 1 | 0 | 1 | 2 | 0 | 0 | 1 | none | none, under treatment | 4.7 | 224.8 | 350 |
| 12 | 2009/4/30 | M | 54 | 2*2*1 | 1 | 0 | 1 | 3 | 0 | 0 | 1 | / | / | 5.1 | 26.1 | / |
| 13 | 2009/5/6 | M | 46 | 6*4*3.5 | 1 | 0 | 0 | ? | 1 | 0 | 1 | none | none | 6.1 | 641.8 | 2.3 |
| 14 | 2009/5/31 | M | 49 | 3.5*3.5*3 | 1 | 0 | 1 | 2 | 0 | 0 | 1 | none | none | 5.2 | 63.9 | >1000 |
| 15 | 2009/6/16 | F | 44 | 2.3*1.8*1.2 | 1 | 0 | 0 | 3 | 1 | 1 | 2 | postoperative 2 month | postoperative 4 month | 5.5 | 430 | 556 |
| 16 | 2009/7/27 | M | 40 | 22*12*6 | 2 | 2 | 0 | 3 | 1 | 1 | 3 | postoperative 2 month | postoperative 4 month | 4.7 | 373.3 | >1000 |
| 17 | 2009/7/29 | M | 63 | 5.5*5.5*4 | 1 | 0 | 1 | 2 | 0 | 0 | 1 | none | none | 7.8 | 36 | 545 |
| 18 | 2009/10/26 | M | 51 | 2.5*2*1 | 1 | 0 | 1 | 1 | 0 | 0 | 1 | none | none | 4.7 | 20.8 | 1.6 |
| 19 | 2009/11/6 | F | 70 | 15*11*9 | 1 | 0 | 0 | 2 | 0 | 0 | 1 | postoperative 11 month | none, under treatment | / | 130.5 | 15.8 |
| 20 | 2009/11/19 | M | 47 | 4*3.5*3.5 | 1 | 0 | 1 | 2 | 0 | 0 | 1 | none | none | 4.8 | 146.2 | 4.1 |
| 21 | 2009/12/10 | M | 53 | 6*5*4 | 1 | 0 | 0 | 3 | 1 | 1 | 3 | postoperative 2 month | postoperative 7 month | 4.9 | 31.7 | 2.4 |
| 22 | 2010/1/11 | M | 60 | 3*3*3; 2*2*2 | 2 | 0 | 1 | 2 | 0 | 0 | 2 | postoperative 4 month | postoperative 22 month | 4.5 | 110.5 | 8.4 |
| 23 | 2010/4/7 | F | 36 | 15*13*8 | 1 | 0 | 1 | 2 | 0 | 0 | 1 | postoperative 10 month | none | 5.4 | 33.1 | 6.3 |
| 24 | 2010/4/9 | M | 39 | 16*9.5*6.5 | 1 | 0 | 0 | 3 | 1 | 1 | 3 | postoperative 3 month | postoperative 7 month | 4.5 | 181 | / |
| 25 | 2010/5/28 | M | 64 | 5*5*5 | 1 | 0 | 1 | 2 | 0 | 0 | 1 | / | / | 7.84 | 55.5 | 57.3 |
| 26 | 2010/6/3 | F | 59 | 6.5*6*5 | 1 | 0 | 1 | 2 | 0 | 0 | 1 | yes | none | 6.2 | 142.3 | 410 |
| 27 | 2010/7/1 | M | 55 | 10.5*7.5*7.5 | 1 | 0 | 1 | 2 | 0 | 0 | 1 | postoperative 13 month | under treatment | 4.1 | 115.7 | 7.7 |
| 28 | 2010/7/14 | F | 66 | 3*2*2 | 1 | 1 | 0 | 2 | 1 | 1 | 3 | none | none | 8.02 | 60.1 | 33.1 |
| 29 | 2010/11/3 | M | 61 | 12.5*8.5*7.5 | 1 | 0 | 1 | 3 | 0 | 0 | 1 | none | none | 5.16 | 29.6 | >1000 |
| 30 | 2010/11/18 | M | 40 | 5*4*3 | 1 | 0 | 1 | 2 | 0 | 0 | 1 | / | / | 4.25 | 36.9 | >1000 |
| 31 | 2010/11/24 | M | 52 | 3.5*3*3 | 1 | 0 | 1 | 2 | 0 | 0 | 1 | none | none | 5.22 | 36.4 | 3.7 |
| 32 | 2010/12/2 | M | 65 | 3*2.5*2; 2*1.5*1 | 2 | 0 | 1 | 3 | 0 | 0 | 2 | postoperative 5 month | none | 11.3 | 41.4 | 91.8 |
| 33 | 2010/12/17 | M | 50 | 6*3*3 | 1 | 1 | / | 2 | 1 | 1 | 3 | postoperative 3 month | postoperative 6 month | / | / | / |
| 34 | 2010/12/24 | M | 47 | 6.5*5.5*5 | 1 | 0 | 1 | 2 | 0 | 0 | 1 | none | none | 4.42 | 42.5 | 4.4 |
| 35 | 2007/3/5 | M | 37 | 7*7*4 | 1 | 0 | 0 | 2 | 1 | 0 | 3 | postoperative 2 month | postoperative 4 month | 4.2 | 119 | 6.75 |
| 36 | 2007/10/15 | M | 71 | 12*8*8cm | 1 | 0 | 0 | 3 | 0 | 0 | 3 | / | postoperative 5 month | 4.9 | 54.5 | 350 |
| 37 | 2007/10/22 | M | 46 | 10.5*10*9.5 | 1 | 0 | 1 | 2 | 0 | 0 | 3 | postoperative 4 month | postoperative 8 month | 3.8 | 144.1 | 350 |
| 38 | 2008/2/28 | M | 56 | 6*6*6 | 1 | 0 | 1 | 3 | 0 | 0 | 1 | postoperative 11 month | postoperative 17 month | 7.7 | 68.1 | 296.5 |
| 39 | 2008/3/14 | M | 47 | 7*5*5 | 2 | 1 | 0 | 2 | 0 | 0 | 1 | / | / | 8.9 | 104.9 | —— |
| 40 | 2008/6/5 | F | 40 | 22*11*7 | 1 | 0 | 0 | 2 | 1 | 1 |  | postoperative 2 month | postoperative 38 month | 5.7 | 267.5 | >1000 |
| 41 | 2008/6/20 | M | 58 | 8*8*4 | 1 | 0 | 0 | 3 | 1 | 1 | 3 | none | none | 5.5 | 196.5 | >1000 |
| 42 | 2008/7/22 | M | 49 | 9*6*4; 2.8*2.5*1 | 2 | 0 | 0 | 3 | 1 | 1 | 3 | postoperative 6 month | postoperative 10 month | 5.5 | 178.4 | 6.8 |
| 43 | 2008/8/4 | M | 35 | 11.5*11*8 | 1 | 0 | 0 | 3 | 1 | 1 | 3 | / | / | 4.2 | 101.3 | 649.44 |
| 44 | 2008/12/15 | M | 70 | 10*8*7 | 1 | 0 | 0 | 2 | 1 | 0 | 3 | postoperative 10 month | postoperative 11 month | 3.9 | 175.2 | 297.2 |
| 45 | 2008/12/25 | M | 68 | 2.5*2.5*2 | 1 | 0 | 1 | 2 | 0 | 0 | 1 | none | none | 4.8 | 12.1 | 8 |
| 46 | 2009/3/6 | M | 54 | 3*2.5*1.7 | 1 | 0 | 1 | 2 | 0 | 0 | 1 | yes | / | 5.7 | 19.3 | 123.2 |
| 47 | 2009/3/16 | F | 67 | 4*4*3 | 1 | 0 | 1 | 2 | 0 | 0 | 1 | none | none | 5 | 21.6 | 350 |
| 48 | 2009/3/18 | M | 45 | 4*3*2.5 | 1 | 0 | 1 | 2 | 0 | 0 | 1 | postoperative 27 month | none, under treatment | 4.9 | 107.1 | 40.4 |
| 49 | 2009/3/25 | M | 34 | 15*6*4 | 1 | 0 | 0 | 2 | 0 | 0 | 1 | none | none | 3.6 | 79.1 | 270 |
| 50 | 2009/3/30 | M | 71 | 2*1.5*1 | 1 | 0 | 1 | 2 | 0 | 0 | 1 | postoperative 21 month | none | 5.3 | 36.7 | 3.5 |
| 51 | 2009/4/29 | M | 63 | 9*9*8.5 | 1 | 0 | 0 | 2 | 0 | 0 | 1 | / | / | 5 | 131.5 | >1000 |
| 52 | 2009/6/23 | M | 31 | 4.5*4*3.5 | 1 | 0 | 1 | 3 | 0 | 0 | 1 | none | none | 4.7 | 133.7 | 143.9 |
| 53 | 2009/9/27 | M | 58 | 15*8*5 | 2 | 1 | 1 | 3 | 1 | 1 | 3 | postoperative 4 month | postoperative 7 month | 5.7 | 209.4 | >1000 |
| 54 | 2009/11/18 | M | 17 | 15*12*10 | 1 | 0 | 1 | 1 | 0 | 0 | 1 | none | none | 3.5 | 196.9 | >1000 |
| 55 | 2010/3/1 | M | 50 | 13*11*8; 3*3*3 | 2 | 0 | 1 | 2 | 1 | 1 | 3 | postoperative 1 month | postoperative 3 month | 11.6 | 174.5 | 38 |
| 56 | 2010/3/16 | M | 68 | 2*1.3*1 | 1 | 0 | 0 | 2 | 0 | 0 | 1 | postoperative 7 month | none | 5.7 | 138.3 | 287 |
| 57 | 2010/4/20 | M | 57 | 6*5*4 | 1 | 1 | 0 | 2 | 0 | 0 | 1 | postoperative 6 month | postoperative 10 month | 9.4 | 267.1 | >1000 |
| 58 | 2010/5/6 | F | 48 | 2.8*2.5*2.5 | 1 | 0 | 1 | 2 | 0 | 0 | 1 | none | none | 5 | 18.4 | 71.8 |
| 59 | 2010/6/2 | M | 56 | 2.7*2.2*2 | 1 | 0 | 1 | 2 | 0 | 0 | 1 | none | none | 7.57 | 154 | 11.7 |
| 60 | 2010/8/5 | M | 44 | 7.5*5*4.5 | 1 | 0 | 1 | 2 | 0 | 0 | 1 | none | none | 5.3 | 75.2 | 74 |
| 61 | 2010/8/23 | F | 42 | 7.5*5.5*4.5cm | 1 | 0 | 0 | 3 | 0 | 0 | 1 | none | none | 5.34 | 12.1 | 18.9 |
| 62 | 2010/9/2 | M | 45 | 4.5*4*4 | 1 | 0 | 1 | 2 | 0 | 0 | 1 | none | none | 4.72 | 21.3 | 396.7 |
| 63 | 2010/9/21 | M | 52 | 7*6*5 | 1 | 0 | 1 | 3 | 1 | 1 | 3 | postoperative 1 month | postoperative 2 month | 5.81 | 48.8 | >1000 |
| 64 | 2010/9/28 | M | 49 | 0.1-8 | 2 | 0 | 0 | 3 | 1 | 1 | 3 | / | / | 5.86 | 171 | >1000 |
| 65 | 2010/9/30 | M | 46 | 5*4*4 | 1 | 0 | 1 | 2 | 0 | 0 | 1 | none | none | 4.46 | 56.7 | 707.2 |
| 66 | 2010/10/22 | M | 65 | ≤6cm | 2 | 0 | 0 | 3 | 1 | 1 | 3 | postoperative 6 month | none | 6.18 | 186.7 | 32.2 |
| 67 | 2004/6/8 | F | 28 | 10*10*9 | 1 | 0 | 0 | 3 | 0 | 0 | 1 | postoperative 32 month | none | 4.78 | 52 | >1000 |
| 68 | 2005/12/15 | M | 60 | 4*4*5 | 1 | 1 | 0 | 3 | 0 | 0 | 1 | postoperative 31 month | postoperative 36 month | 4.97 | 167 | 8.9 |
| 69 | 2006/2/20 | M | 70 | 2*2*2 | 1 | 1 | 0 | 2 | 0 | 0 | 1 | postoperative 43 month | none | 5.96 | 13 | 7.4 |
| 70 | 2006/3/8 | M | 51 | 5*5*3 | 1 | 1 | 0 | 3 | 0 | 0 | 1 | none | none | 4.86 | 178 | >1000 |
| 71 | 2006/3/27 | M | 38 | 2*2*2 | 1 | 1 | 0 | 3 | 0 | 0 | 1 | postoperative 42 month | none | 4.72 | 30 | >1000 |
| 72 | 2006/3/26 | F | 48 | 4*3 | 1 | 1 | 0 | 2 | 0 | 0 | 1 | none | none | 5.32 | 44 | 72.5 |
| 73 | 2006/5/15 | F | 66 | 3*3*3 | 1 | 0 | 1 | 3 | 0 | 0 | 1 | none | postoperative 12 month | 4.39 | 75 | >1000 |
| 74 | 2006/5/18 | M | 41 | 4*4*4 | 1 | 1 | 0 | 3 | 0 | 0 | 1 | postoperative 3 month | postoperative 11 month | 4.95 | 35 | 738 |
| 75 | 2006/5/25 | M | 31 | 3*3*2 | 1 | 0 | 0 | 3 | 0 | 0 | 1 | none | none | 4.71 | 35 | >1000 |
| 76 | 2006/6/9 | M | 42 | 6*5.5*4.5 | 1 | 1 | 0 | 3 | 0 | 0 | 1 | postoperative 3 month | none | 5.18 | 53 | 14.4 |
| 77 | 2006/11/16 | M | 58 | 6*5*5 | 1 | 1 | 0 | 3 | 0 | 0 | 1 | postoperative 12 month | postoperative 35 month | 4.37 | 138 | 5.6 |
| 78 | 2006/12/14 | M | 64 | 4*4*3 | 1 | 1 | 0 | 3 | 0 | 0 | 1 | postoperative 6 month | postoperative 30 month | 4.98 | 56 | 295 |
| 79 | 2006/12/18 | M | 62 | 4*5 | 1 | 0 | 0 | 3 | 0 | 0 | 1 | postoperative 14 month | postoperative 20 month | 5.19 | 80 | 14.6 |
| 80 | 2006/12/28 | M | 49 | 2*2*1 | 1 | 1 | 0 | 3 | 0 | 0 | 1 | none | none | 4.19 | 20 | 7.5 |
| 81 | 2007/1/3 | M | 41 | 5*5 | 1 | 0 | 0 | 3 | 0 | 0 | 1 | postoperative 7 month | postoperative 38 month | 4.95 | 61 | 2.9 |
| 82 | 2007/1/18 | F | 53 | 3*3*2 | 1 | 0 | 0 | 2 | 0 | 0 | 1 | none | none | 4.29 | 10 | 161.9 |
| 83 | 2007/2/2 | M | 42 | 4*4 | 1 | 0 | 0 | 3 | 1 | 0 | 1 | postoperative 8 month | postoperative 29 month | 4.62 | 48 | >1000 |
| 84 | 2007/1/31 | M | 49 | 4*3*3 | 1 | 0 | 0 | 3 | 0 | 0 | 1 | none | none | 4.66 | 43 | >1000 |
| 85 | 2007/3/8 | M | 57 | 6*5*5 | 1 | 0 | 0 | 3 | 0 | 0 | 1 | postoperative 6 month | postoperative 18 month | 7.4 | 302 | 288.5 |
| 86 | 2007/3/27 | M | 49 | 2*2 | 1 | 1 | 0 | 3 | 0 | 0 | 1 | none | none | 5.76 | 154 | 126.7 |
| 87 | 2007/3/28 | M | 47 | 3*3*3 | 1 | 1 | 0 | 3 | 0 | 0 | 1 | none | none | 4.55 | 22 | >1000 |
| 88 | 2007/4/5 | M | 39 | 4*3*3 | 1 | 0 | 0 | 3 | 0 | 0 | 1 | none | none | 4.6 | 67 | >1000 |
| 89 | 2007/4/27 | M | 66 | 4*4*3 | 1 | 0 | 0 | 3 | 0 | 0 | 1 | postoperative 7 month | none | 5.56 | 76 | >1000 |
| 90 | 2007/4/30 | M | 48 | 6*4*3 | 1 | 0 | 0 | 3 | 1 | 1 | 1 | postoperative 2 month | postoperative 9 month | 5.07 | 116 | >1000 |
| 91 | 2007/5/10 | M | 52 | 5*4 | 2 | 0 | 0 | 3 | 0 | 0 | 1 | postoperative 26 month | none | 4.35 | 122 | 8.2 |
| 92 | 2007/5/31 | M | 52 | 5.5*4*4 | 1 | 1 | 0 | 3 | 0 | 0 | 1 | postoperative 14 month | postoperative 28 month | 8 | 23 | 40.5 |
| 93 | 2007/6/13 | F | 54 | 3*2.5 | 1 | 0 | 0 | 3 | 0 | 0 | 1 | none | none | 4.39 | 18 | 39.3 |
| 94 | 2007/6/14 | M | 62 | 4*4*4 | 1 | 0 | 0 | 2 | 0 | 0 | 1 | postoperative 10 month | postoperative 22 month | 3.86 | 61 | 20.26 |
| 95 | 2007/6/19 | M | 67 | 4*4*3 | 1 | 0 | 0 | 2 | 0 | 0 | 1 | none | none | 6.46 | 51 | 10.3 |
| 96 | 2007/7/10 | M | 53 | 3*3*3 | 1 | 1 | 0 | 2 | 0 | 0 | 1 | postoperative 40 month | none | 6.06 | 60 | 112.4 |
| 97 | 2007/7/16 | M | 54 | 3*3*2 | 1 | 0 | 0 | 2 | 0 | 0 | 1 | none | none | 4.86 | 28 | 2.6 |
| 98 | 2007/7/30 | M | 36 | 5*4*3 | 1 | 1 | 0 | 3 | 0 | 0 | 1 | none | none | 3.98 | 60 | 11.9 |
| 99 | 2007/8/13 | M | 42 | 5*5*4 | 1 | 1 | 0 | 3 | 0 | 0 | 1 | none | none | 4.01 | 28 | >1000 |
| 100 | 2007/8/10 | M | 49 | 2.5*2.5*2 | 1 | 0 | 0 | 2 | 0 | 0 | 1 | none | none | 4.17 | 30 | 3.7 |
| 101 | 2007/8/29 | M | 41 | 3*3*2 | 1 | 0 | 0 | 3 | 0 | 0 | 1 | none | none | 4.27 | 19 | 93 |
| 102 | 2007/9/18 | M | 70 | 4*3*3 | 1 | 1 | 0 | 3 | 0 | 0 | 1 | postoperative 13 month | postoperative 38 month | 5.37 | 38 | >1000 |
| 103 | 2007/9/18 | M | 58 | 4*4*3 | 1 | 0 | 0 | 2 | 0 | 0 | 1 | postoperative 23 month | none | 4.64 | 131 | 16 |
| 104 | 2007/9/20 | M | 50 | 1。5*1.5*1 | 1 | 0 | 0 | 2 | 0 | 0 | 1 | postoperative 24 month | none | 4.67 | 145 | 5.2 |
| 105 | 2007/11/22 | M | 50 | 4*3*3 | 1 | 0 | 0 | 3 | 0 | 0 | 1 | postoperative 9 month | postoperative 40 month | 4.86 | 32 | >1000 |
| 106 | 2007/11/7 | M | 42 | 4*4*4 | 1 | 0 | 0 | 3 | 0 | 0 | 1 | none | none | 5.09 | 110 | 374.1 |
| 107 | 2007/11/15 | M | 55 | 2*1.5*1.5 | 1 | 0 | 0 | 3 | 0 | 0 | 1 | none | none | 4.81 | 30 | 4.7 |
| 108 | 2007/11/21 | M | 73 | 6*6*5 | 1 | 1 | 0 | 2 | 0 | 0 | 1 | none | none | 4.49 | 113 | 2.3 |
| 109 | 2007/11/22 | M | 55 | 2*1.5*1.5 | 1 | 0 | 0 | 2 | 0 | 0 | 1 | none | none | 5.06 | 40 | 1.2 |
| 110 | 2007/11/27 | M | 46 | 1.5*1.5*1 | 1 | 0 | 0 | 3 | 0 | 0 | 1 | none | none | 4.91 | 32 | 527.8 |
| 111 | 2007/12/3 | M | 46 | 4.5*4*3 | 1 | 1 | 0 | 3 | 0 | 0 | 1 | none | none | 6.88 | 68 | 4.7 |
| 112 | 2007/12/5 | M | 39 | 3*3*3 | 1 | 0 | 0 | 3 | 0 | 0 | 1 | none | none | 4.23 | 109 | >1000 |
| 113 | 2007/12/6 | F | 55 | 4*4*3 | 1 | 1 | 0 | 3 | 0 | 0 | 1 | postoperative 12 month | postoperative 42 month | 4.47 | 88 | >1000 |
| 114 | 2007/12/19 | M | 68 | 3*3*2 | 1 | 0 | 0 | 2 | 0 | 0 | 1 | none | none | 4.57 | 47 | 2.6 |
| 115 | 2007/12/27 | M | 48 | 3*3*3 | 1 | 1 | 0 | 2 | 0 | 0 | 1 | postoperative 13 month | none, under treatment | 5.11 | 78 | 492.2 |
| 116 | 2008/1/7 | M | 46 | 5*5*5 | 1 | 1 | 0 | 2 | 0 | 0 | 1 | postoperative 12 month | postoperative 39 month | 4.65 | 241 | 44 |
| 117 | 2008/1/22 | M | 56 | 11*10*9 | 1 | 1 | 0 | 2 | 0 | 0 | 1 | / | postoperative 43 month | 4.96 | 104 | 21.5 |
| 118 | 2008/3/12 | M | 63 | 2*2*2 | 1 | 1 | 0 | 2 | 0 | 0 | 1 | postoperative 13 month | none | 4.32 | 112 | 310 |
| 119 | 2008/3/10 | M | 43 | 5*5*4 | 1 | 1 | 0 | 3 | 0 | 0 | 1 | postoperative 19 month | postoperative 25 month | 5.59 | 98 | 6.7 |
| 120 | 2008/3/18 | M | 32 | 8*7*6 | 1 | 0 | 0 | 3 | 0 | 0 | 1 | postoperative 5 month | postoperative 16 month | 4.1 | 75 | >1000 |
| 121 | 2008/3/18 | M | 60 | 5*4*3 | 1 | 0 | 0 | 3 | 0 | 0 | 1 | postoperative 9 month | postoperative 41 month | 6.09 | 146 | 892.8 |
| 122 | 2008/3/25 | M | 46 | 1.5*1.5*1 | 1 | 1 | 0 | 3 | 0 | 0 | 1 | none | none | 7.17 | 43 | 4 |
| 123 | 2008/3/20 | F | 41 | 11*10*8 | 1 | 0 | 0 | 3 | 0 | 0 | 1 | none | none | 4.41 | 46 | >1000 |
| 124 | 2008/3/24 | M | 52 | 5*5*3 | 1 | 0 | 0 | 2 | 0 | 0 | 1 | postoperative 36 month | none | 4.42 | 277 | 9.5 |
| 125 | 2008/3/21 | M | 70 | 3*3*2.5 | 1 | 0 | 0 | 3 | 0 | 0 | 1 | none | none | 5.72 | 58 | 25.8 |
| 126 | 2008/4/2 | M | 70 | 5*5*3 | 1 | 1 | 0 | 3 | 0 | 0 | 1 | none | none | 5.41 | 728 | 6.7 |
| 127 | 2008/4/15 | M | 47 | 8*6*6 | 1 | 1 | 0 | 3 | 0 | 0 | 1 | none | none | 5.92 | 35 | >1000 |
| 128 | 2008/5/21 | M | 59 | 7*6*6 | 1 | 1 | 0 | 2 | 0 | 0 | 1 | none | none | 5.64 | 106 | 2.1 |
| 129 | 2008/5/13 | M | 39 | 6*6*5 | 1 | 0 | 0 | 2 | 0 | 0 | 1 | none | none | 4.84 | 62 | 35.7 |
| 130 | 2008/5/14 | F | 47 | 7*6*6 | 1 | 1 | 0 | 3 | 0 | 0 | 1 | postoperative 29 month | none | 11.3 | 20 | >1000 |
| 131 | 2008/5/19 | M | 31 | 2*1*1 | 1 | 0 | 0 | 3 | 0 | 0 | 1 | none | none | 4.2 | 32 | 485.7 |
| 132 | 2008/5/28 | M | 37 | 5*5*4 | 1 | 1 | 0 | 2 | 0 | 0 | 1 | postoperative 9 month | postoperative 26 month | 6 | 63 | 161.3 |
| 133 | 2008/6/11 | M | 31 | 6*5*4 | 1 | 1 | 0 | 3 | 0 | 0 | 1 | none | none | 4.16 | 64 | 2.4 |
| 134 | 2008/7/4 | M | 43 | 6*6*4 | 1 | 1 | 0 | 3 | 0 | 0 | 1 | postoperative 3 month | postoperative 10 month | 4.82 | 53 | >1000 |
| 135 | 2008/7/4 | M | 45 | 9*8*8 | 1 | 0 | 0 | 3 | 0 | 0 | 1 | postoperative 2 month | none | 6.23 | 131 | >1000 |
| 136 | 2008/7/16 | M | 64 | 3*3*2 | 1 | 1 | 0 | 3 | 0 | 0 | 1 | none | none | 4.07 | 12 | 1.3 |
| 137 | 2008/7/23 | M | 46 | 12*10*9 | 1 | 0 | 0 | 3 | 0 | 0 | 1 | / | postoperative 32 month | 4.6 | 112 | 895 |
| 138 | 2008/7/28 | M | 70 | 10*7*6 | 1 | 0 | 0 | 3 | 0 | 0 | 1 | postoperative 19 month | postoperative 40 month | 10.7 | 89 | >1000 |
| 139 | 2008/7/29 | M | 59 | 7*6*5 | 1 | 1 | 0 | 2 | 0 | 0 | 1 | postoperative 9 month | postoperative 35 month | 6.94 | 143 | 6.9 |
| 140 | 2006/7/11 | M | 51 | 2*2 | 1 | 0 | 0 | 3 | 0 | 0 | 1 | none | none | 4.2 | 26 | 318.3 |
| 141 | 2007/1/9 | M | 54 | 4*4 | 1 | 0 | 0 | 3 | 0 | 0 | 1 | none | none | 4.59 | 110 | 107.5 |
| 142 | 2007/10/4 | F | 29 | 1*1*1 | 1 | 1 | 0 | 2 | 0 | 0 | 1 | none | none | 4.59 | 15 | 799.1 |
| 143 | 2005/6/22 | M | 64 | 2*1; 1*1 | 2 | 1 | 0 | 2 | 0 | 0 | 2 | postoperative 11 month | postoperative 32 month | 4.76 | 617 | 825 |
| 144 | 2007/4/25 | M | 46 | 2*2*2; 1.5*1.5*1 | 2 | 0 | 0 | 3 | 1 | 1 | 2 | postoperative 7 month | postoperative 15 month | 66.5 | 384 | 21 |
| 145 | 2007/7/2 | F | 53 | 4*4*3 | 1 | 0 | 1 | 3 | 1 | 1 | 2 | none | none | 12.2 | 152 | 2.4 |
| 146 | 2007/9/7 | M | 62 | 5*3*2.5 | 2 | 0 | 0 | 3 | 0 | 0 | 2 | postoperative 3 month | postoperative 26 month | 4.38 | 52 | 8.7 |
| 147 | 2007/11/9 | M | 57 | 4.5*4*4; 2.5*2*2; 2*1*1 | 2 | 1 | 0 | 3 | 0 | 0 | 2 | postoperative 2 month | postoperative 12 month | 4.37 | 90 | 35.3 |
| 148 | 2007/11/13 | M | 50 | 8*8*6 | 1 | 0 | 1 | 3 | 0 | 0 | 2 | postoperative 7 month | none | 4.39 | 54 | 18.8 |
| 149 | 2007/12/3 | M | 60 | 7*6*6 | 1 | 0 | 1 | 3 | 0 | 0 | 2 | postoperative 8 month | none | 4.85 | 81 | 19.9 |
| 150 | 2007/12/6 | M | 64 | 13*10*9 | 1 | 0 | 0 | 3 | 1 | 0 | 2 | postoperative 2 month | postoperative 7 month | 4.15 | 188 | >1000 |
| 151 | 2008/3/3 | M | 41 | 13*10*9 | 1 | 1 | 0 | 2 | 1 | 0 | 2 | none | none | 4.68 | 158 | 3.5 |
| 152 | 2008/4/8 | M | 55 | 5*5*4 | 2 | 1 | 0 | 3 | 0 | 0 | 2 | postoperative 4 month | postoperative 11 month | 4.78 | 27 | >1000 |
| 153 | 2008/4/17 | M | 52 | 3*3*3 | 1 | 1 | 1 | 2 | 0 | 0 | 2 | postoperative 24 month | none | 7.57 | 43 | 23.2 |
| 154 | 2008/5/6 | M | 49 | 6*5*5; 0.5*0.5 | 2 | 1 | 1 | 3 | 0 | 0 | 2 | none | none | 8.7 | 297 | 6 |
| 155 | 2008/4/23 | M | 73 | 3*2.5*2.5 | 1 | 0 | 1 | 3 | 0 | 0 | 2 | postoperative 10 month | postoperative 30 month | 4.81 | 163 | 11.6 |
| 156 | 2008/4/30 | M | 56 | 4*4*3; 1*1*1 | 2 | 0 | 1 | 2 | 1 | 0 | 2 | postoperative 21 month | none | 4.77 | 62 | 3.6 |
| 157 | 2008/5/9 | F | 47 | 6.6*5*5 | 2 | 1 | 0 | 3 | 0 | 0 | 2 | postoperative 7 month | none | 4.35 | 27 | >1000 |
| 158 | 2008/7/8 | M | 70 | 3*2; 1.5*1*1 | 2 | 0 | 1 | 3 | 0 | 0 | 2 | / | postoperative 40 month | 4.97 | 26 | 2.3 |
| 159 | 2008/8/11 | F | 44 | 5.5*4*3 | 1 | 1 | 0 | 3 | 0 | 0 | 2 | postoperative 2 month | none | 4.98 | 187 | >1000 |
| 160 | 2006/3/23 | M | 56 | 10*9*9 | 1 | 0 | 0 | 3 | 1 | 0 | 3 | postoperative 18 month | postoperative 23 month | 5.68 | 109 | 50 |
| 161 | 2006/4/13 | M | 61 | 7*6*6 | 1 | 0 | 1 | 3 | 1 | 1 | 3 | postoperative 3 month | none | 5.12 | 4.6 | 754.9 |
| 162 | 2006/5/31 | M | 51 | 5.5*5*5 | 1 | 0 | 0 | 3 | 1 | 1 | 3 | postoperative 4 month | postoperative 10 month | 4.38 | 57 | 176 |
| 163 | 2006/6/2 | M | 70 | 7*7*5 | 1 | 0 | 1 | 3 | 1 | 1 | 3 | / | postoperative 3 month | 6.36 | 285 | 14.1 |
| 164 | 2006/6/28 | M | 54 | 13*12 | 1 | 0 | 1 | 3 | 1 | 1 | 3 | postoperative 5 month | postoperative 10 month | 4.27 | 126 | >1000 |
| 165 | 2006/6/26 | M | 48 | 6*6*5 | 1 | 1 | 0 | 3 | 1 | 1 | 3 | postoperative 36 month | postoperative 60 month | 5.1 | 541 | 169.5 |
| 166 | 2006/11/10 | M | 49 | 8*7*6 | 1 | 0 | 1 | 3 | 0 | 0 | 3 | postoperative 6 month | postoperative 27 month | 4.31 | 50 | 548.6 |
| 167 | 2006/11/21 | M | 45 | 3*3*1 | 1 | 0 | 1 | 3 | 1 | 1 | 3 | postoperative 31 month | none | 9.61 | 54 | >1000 |
| 168 | 2007/3/19 | M | 40 | 12*10*9 | 1 | 0 | 0 | 3 | 1 | 1 | 3 | postoperative 2 month | postoperative 5 month | 3.68 | 177 | >1000 |
| 169 | 2007/3/22 | F | 62 | 10*9*9 | 1 | 0 | 1 | 3 | 1 | 1 | 3 | postoperative 2 month | postoperative 4 month | 7.88 | 227 | >1000 |
| 170 | 2007/4/5 | M | 46 | 6*6*5; 1*0.6*0.6 | 1 | 1 | 1 | 3 | 0 | 0 | 3 | postoperative 5 month | postoperative 12 month | 5.23 | 59 | 9.8 |
| 171 | 2007/4/16 | M | 51 | 6*5*5 | 1 | 1 | 0 | 2 | 1 | 1 | 3 | postoperative 2 month | postoperative 4 month | 4.58 | 97 | >1000 |
| 172 | 2007/6/28 | M | 45 | 8*8*7 | 1 | 0 | 1 | 3 | 0 | 0 | 3 | postoperative 6 month | postoperative 43 month | 4.44 | 119 | 103.7 |
| 173 | 2007/7/13 | M | 54 | 4*4*3 | 1 | 0 | 0 | 3 | 1 | 1 | 3 | postoperative 20 month | postoperative 48 month | 4.1 | 64 | 41.4 |
| 174 | 2007/7/10 | M | 58 | 19*16*10 | 2 | 0 | 1 | 3 | 1 | 1 | 3 | postoperative 3 month | postoperative 9 month | 6.1 | 114 | >1000 |
| 175 | 2007/7/25 | M | 56 | 5*4*4 | 1 | 1 | 1 | 2 | 1 | 1 | 3 | none | none | 4.66 | 48 | 1.4 |
| 176 | 2007/8/2 | M | 38 | 22*15*12; 1*1*1 | 1 | 1 | 1 | 3 | 1 | 0 | 3 | postoperative 2 month | postoperative 4 month | 4.52 | 202 | >1000 |
| 177 | 2007/8/16 | F | 47 | 10*8*6 | 1 | 0 | 1 | 3 | 0 | 0 | 3 | postoperative 9 month | postoperative 20 month | 4.02 | 17 | >1000 |
| 178 | 2007/8/24 | M | 58 | 12*10*9 | 2 | 0 | 1 | 3 | 1 | 1 | 3 | postoperative 3 month | postoperative 15 month | 6.14 | 159 | 463.5 |
| 179 | 2007/8/24 | M | 49 | 8*8*6 | 1 | 0 | 0 | 3 | 1 | 1 | 3 | postoperative 6 month | postoperative 12 month | 4.91 | 33 | 512.4 |
| 180 | 2007/9/19 | M | 46 | 12*10*10 | 1 | 0 | 0 | 3 | 1 | 1 | 3 | postoperative 5 month | postoperative 9 month | 4.73 | 187 | 52.5 |
| 181 | 2007/10/31 | M | 32 | 6*5*5 | 1 | 0 |  | 3 | 1 | 1 | 3 | postoperative 6 month | postoperative 10 month | 3.94 | 93 | 631.7 |
| 182 | 2007/12/13 | M | 54 | 10*9*7 | 1 | 0 | 0 | 3 | 1 | 1 | 3 | postoperative 3 month | postoperative 12 month | 6.93 | 129 | >1000 |
| 183 | 2008/1/23 | M | 52 | 7*7*6; 1*1*1 | 2 | 1 | 0 | 3 | 0 | 0 | 3 | postoperative 17 month | postoperative 20 month | 4.38 | 115 | 12.4 |
| 184 | 2008/2/27 | M | 48 | 11*11*10; 1*1*0.8 | 2 | 1 | 1 | 3 | 1 | 1 | 3 | postoperative 2 month | postoperative 7 month | 4.84 | 173 | >1000 |
| 185 | 2008/3/14 | F | 43 | 10*7*7; 5*3*3 | 2 | 0 | 0 | 3 | 1 | 1 | 3 | postoperative 9 month | none, under treatment | 4.77 | 139 | >1000 |
| 186 | 2008/3/24 | M | 36 | 6.5*5*4; 4.5*3*3 | 2 | 0 | 0 | 3 | 1 | 0 | 3 | postoperative 5 month | postoperative 8 month | 5.86 | 91 | >1000 |
| 187 | 2008/5/14 | M | 46 | 7*7*6 | 2 | 0 | 0 | 3 | 0 | 0 | 3 | postoperative 2 month | postoperative 9 month | 4.66 | 178 | >1000 |
| 188 | 2008/5/14 | M | 59 | 4*4*4 2*2*2 | 2 | 0 | 0 | 3 | 1 | 1 | 3 | postoperative 3 month | postoperative 10 month | 4.06 | 97 | 27.54 |
| 189 | 2008/5/15 | M | 53 | 4*4*3 | 2 | 0 | 0 | 3 | 1 | 0 | 3 | postoperative 2 month | postoperative 11 month | 5.7 | 268 | 518.2 |
| 190 | 2008/7/16 | F | 43 | 14*13*10 | 1 | 1 | 0 | 3 | 0 | 0 | 3 | postoperative 2 month | postoperative 11 month | 4.66 | 161 | >1000 |
